# Inactivation of cellular retinol-binding protein 1 protects against bis-retinoid accumulation and light-induced retinal degeneration in mice

**DOI:** 10.1016/j.jbc.2025.110538

**Published:** 2025-07-30

**Authors:** Made Airanthi K. Widjaja-Adhi, Jaclyn Swigris, Jacqueline Plau, Chloe Chung, Anna Walczak-Szeffer, Beata Jastrzebska, William S. Blaner, Marcin Golczak

**Affiliations:** 1 Department of Pharmacology, School of Medicine, Case Western Reserve University, Cleveland, Ohio, USA; 2 Department of Cell Cultures and Genomic Analysis, Medical University of Lodz, Lodz, Poland; 3 Cleveland Center for Membrane and Structural Biology, School of Medicine, Case Western Reserve University, Cleveland, Ohio, USA; 4 Department of Medicine, College of Physicians and Surgeons, Columbia University, New York, New York, USA

**Keywords:** retinol-binding protein 1, visual cycle, A2E, retinoids, retinal degeneration, RBP1

## Abstract

Delayed clearance of all-*trans*-retinal (at-RAL) in photoreceptors is linked to prevalent retinal diseases such as Stargardt disease, rod-cone dystrophies, and age-related macular degeneration. Pharmacological modulation of retinoid metabolism in the eye presents a promising therapeutic strategy, with cellular retinol-binding protein 1 (RBP1) emerging as a potential target. However, it lacks genetic validation as a therapeutic target in an animal model of human disease. Thus, we investigated the effect of the *Rbp1* gene inactivation on the phenotype of the *Abca4*^*−/−*^*/Rdh8*^*−/−*^ model of Stargardt disease. Triple knockout (*Abca4*^*−/−*^*/Rdh8*^*−/−*^*/Rbp1*^*−/−*^) mice were protected against light-induced retinal degeneration. RBP1 deficiency also slowed bis-retinoid accumulation in aged animals. Furthermore, pharmacological inhibition of RBP1 alleviated the retinal degeneration phenotype. These findings provide both genetic and pharmacological evidence supporting RBP1 as a promising therapeutic target for retinal degenerative diseases associated with impaired all-*trans*-retinal clearance.

Perception of light is a complex biochemical process that converts the energy of photons into neuronal signals (1, 2). It begins with the absorption of light by visual pigments in photoreceptor cells in the retina, causing a photoisomerization of visual pigment chromophore, 11-*cis*-retinal (11c-RAL), and triggering a cascade of phototransduction events that ultimately convert light into a neural graded potential (3). Central to sustaining visual perception is the regeneration of the visual chromophore (4). In vertebrates, this regeneration process occurs through a metabolic pathway known as the visual (retinoid) cycle, which takes place in photoreceptor and retinal pigment epithelium (RPE) cells, supporting the opsins of both rods and cones (Fig. 1*A*).

**Figure 1:**
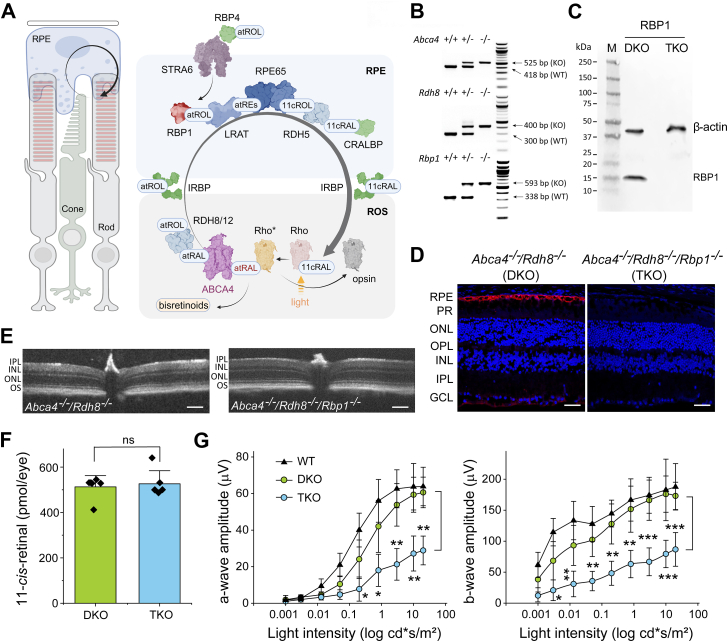
**The role of RBP1 in the visual cycle and generation of *Abca4*^*−/−*^*/Rdh8*^*−/−*^*/Rbp1*^*−/−*^ mouse** l**ines.***A*, schematic representation of the visual cycle, illustrating the cellular and molecular components involved in visual chromophore regeneration. Proteins are color-coded based on their roles: enzymes (*blue*), transporters and receptors (*purple*), and retinoid carriers (*green*). Rhodopsin is shown in *gray*, *pink*, and *yellow* depending on its state, while RBP1 is highlighted in *red*. *B*, representative agarose gels showing genotyping results for *Abca4*^*−/−*^*/Rdh8*^*−/−*^*/Rbp1*^*−/−*^ (TKO) mice and their heterozygous and WT counterparts. *C*, western blot analysis of ocular tissues from *Rbp1*^*−/−*^ mice. Unlike in *Abca4*^*−/−*^*/Rdh8*^*−/−*^ (DKO) controls, RBP1 is absent in eyeball homogenates of *Abca4*^*−/−*^*/Rdh8*^*−/−*^*/Rbp1*^*−/−*^ (TKO) mice. *D*, immunofluorescence-based confirmation of RBP1 deficiency. Retina sections were probed with an anti-RBP1 antibody, revealing specific staining exclusively in the RPE cell layer of *Abca4*^*−/−*^*/Rdh8*^*−/−*^ mice. No immunofluorescence signal was detected in the corresponding *Rbp1*^*−/−*^ mice on the *Abca4*^*−/−*^*/Rdh8*^*−/−*^ (DKO) genetic background. The scale bar represents 50 μm. *E*, the retinal morphology of 4-month-old mice showed no signs of spontaneous retinal degeneration in either mouse line. The scale bar represents 150 μm. PR (photoreceptors), ONL (outer nuclear layer), OPL (outer plexiform layer), INL (inner nuclear layer), IPL (inner plexiform layer); GCL (ganglion cell layer). *F*, quantification of 11c-RAL in the retinas of *Abca4*^*−/−*^*/Rdh8*^*−/−*^ (DKO) and *Abca4*^*−/−*^*/Rdh8*^*−/−*^*/Rbp1*^*−/−*^ (TKO) mice. No statistically significant (ns) differences were observed in visual chromophore content, indicating comparable levels of rhodopsin (n = 6). *G*, the effect of RBP1 deficiency on the rate of dark adaptation. Scotopic ERG responses were recorded after 90 min of dark adaptation following exposure to a bright light flash that bleached ∼80% of the rod visual pigment. Delayed dark adaptation in *Abca4*^*−/−*^*/Rdh8*^*−/−*^*/Rbp1*^*−/−*^ (TKO) mice was evidenced by reduced ERG a- and b-wave amplitudes compared to *Abca4*^*−/−*^*/Rdh8*^*−/−*^ (DKO) mouse line. The absence of RDH8 and ABCA4 alone had a detectable, but much subtler effect on the regeneration of visual pigment, as observed for *Abca4*^*+/+*^*/Rdh8*^*+/+*^*/Rbp1*^*+/+*^ mice, indicated as “WT”. Data represent mean ± S.D. (n = 6). Student’s *t*-test was used for two-group comparisons of individual experimental points (∗*p* < 0.05; ∗∗*p* < 0.005; ∗∗∗*p* < 0.0005). 11c-RAL, 11-cis-retinal; ERG, electroretinogram; RBP1, cellular retinol-binding protein 1; RPE, retinal pigment epithelium.

Dysregulation of the visual cycle can cause or promote the progression of certain retinopathies, including Stargardt disease type 1 (STGD1), light-induced retinopathies, and possibly age-related macular degeneration (2, 5). These retinal degenerative conditions, despite their diverse etiologies, share a common pathological mechanism related to the delayed clearance of all-*trans*-retinal (at-RAL) (6). The resulting prolonged increase in the steady-state concentration of at-RAL leads to cytotoxicity and the accumulation of byproducts of its chemical reactivity, specifically bis-retinoids (*e.g.*, *N*-retinylidene-*N*-retinylethanolamine, A2E, and retinal dimer, among others) (7, 8, 9).

The widely accepted strategy for preventing retinal degenerative diseases associated with diminished at-RAL clearance is to reduce the metabolic flux of retinoids through the visual cycle (2, 10, 11). This concept was first supported by the observation that mice expressing the RPE65 Leu450/Met variant are protected against light-induced retinal degeneration, an effect attributed to slower regeneration of the visual chromophore (12). Subsequent studies indicated that pharmacological intervention in the visual cycle with 13-*cis*-retinoic acid delayed dark adaptation and provided protection against light-induced retinal damage (13). In addition, a slower rate of visual chromophore regeneration was associated with a reduced rate of bis-retinoid accumulation in mice (14).

Based on these observations, several therapeutic strategies have been proposed, including developing specific visual cycle modulators (15, 16, 17, 18). These pharmacological agents are designed to intervene in the visual cycle to slow the regeneration of the visual chromophore by targeting enzymes and proteins involved in *cis*-retinoids regeneration and transport. Early strategies aimed at suppressing the formation or expediting the removal of at-RAL and its derivatives included direct inhibition of retinal pigment epithelium-specific protein 65kDA (RPE65) (19, 20), diminishing ocular vitamin A uptake using serum retinol-binding protein (RBP4) antagonists (21), at-RAL sequestration (22), and administration of deuterated vitamin A derivatives (23). However, despite their conceptual promise, these approaches have several limitations that might hinder their clinical use, including unfavorable pharmacokinetics, excessive suppression of rod function, induction of color vision abnormalities, potential induction of systemic vitamin A deficiency, and the need for a specialized diet low in naturally occurring vitamin A and its precursors (18).

Based on previous research and ongoing clinical trials, we hypothesized the existence of an alternative biological target within the retinoid cycle whose pharmacological modulation could provide therapeutic benefits without the major side effects characteristic of current strategies. To this end, we aimed to validate cellular retinol-binding protein 1 (RBP1, also referred to as CRBP1) as a promising target for controlling ocular retinoid metabolism. RBP1 is a key retinoid-binding protein in the RPE, regulating the intracellular transport of all-*trans*-retinol (at-ROL) from the circulation *via* stimulated by retinoic acid 6 (STRA6)-dependent uptake or its recycling from light-exposed photoreceptors (24) (Fig. 1*A*). The selection of this protein was based on previous reports showing a twofold decrease in the dark adaptation rate in *Rbp1*^*−/−*^ mice compared to WT counterparts (25). Importantly, this slower regeneration of 11c-RAL was not accompanied by spontaneous retinal degeneration. Furthermore, RBP1-deficient mice remain overall healthy when maintained on a vitamin A-sufficient diet, minimizing potential side effects associated with the inhibition of this protein (26).

In our previous publications, we described the discovery of RBP1 inhibitors and provided the mechanistic basis for their selectivity, potency, and *in vivo* effect on the rate of visual chromophore regeneration (27, 28). Thus, we proposed that RBP1 is a potential therapeutic target for treating ocular diseases associated with delayed at-RAL clearance. In this report, we provide the genetic proof-of-concept by examining the consequences of the inactivation of the *Rbp1* gene in an animal model of STGD1. For this purpose, we used *Abca4*^*−/−*^*/Rdh8*^*−/−*^ double KO mice, known for their light sensitivity, acute retinopathies, and excessive accumulation of A2E compared to WT mice. Cross-breeding these mice with the *Rbp1*^*−/−*^ line resulted in *Abca4*^*−/−*^*/Rdh8*^*−/−*^*/Rbp1*^*−/−*^ triple KO offspring that were largely protected against light-induced retinal degeneration. The absence of RBP1 also slowed the accumulation of A2E, leading to significantly fewer retinal condensation products in aged animals. Concurrently with the genetic inactivation of the *Rbp1* gene, pharmacological modulation of RBP1 activity with abnormal cannabidiol (abn-CBD) and other nonretinoid inhibitors provided substantial protection against retinal degeneration in *Abca4*^*−/−*^*/Rdh8*^*−/−*^ mice. Collectively, our findings offer both genetic and pharmacological evidence supporting RBP1 as a promising new biological target for treating retinal diseases associated with decreased at-RAL clearance.

## Results

### Deletion of the *Rbp1* gene protects against acute light-induced retinal degeneration

In our previous studies, we showed that inhibition of RBP1 with abn-CBD was efficacious in preventing light-induced retinal degeneration in Balb/cJ albino mice (27). However, proper validation of RBP1 as a therapeutic target against retinal degeneration requires genetic proof-of-concept in a relevant model of human conditions. For this purpose, we utilized *Abca4*^*−/−*^*/Rdh8*^*−/−*^ mice (29), an animal model of STGD1, to generate an *Abca4*^*−/−*^*/Rdh8*^*−/−*^*/Rbp1*^*−/−*^ mouse line. The deletion of the *Rbp1* gene in the *Abca4*^*−/−*^*/Rdh8*^*−/−*^ line was confirmed by genotyping (Fig. 1*B*), while the absence of RBP1 protein in the eye was verified by western blotting and immunofluorescence (Fig. 1, *C* and *D*). Unlike in *Abca4*^*−/−*^*/Rdh8*^*−/−*^ controls, RBP1 is absent in eyeball homogenates of *Abca4*^*−/−*^*/Rdh8*^*−/−*^*/Rbp1*^*−/−*^ mice. Concurrently, retina sections were probed with an anti-RBP1 antibody, revealing specific staining in the RPE cell layer of *Rbp1*^*+/+*^ mice (Fig. S1). There was no definitive evidence for RBP1 elsewhere in the retina except for a slight fluorescence signal in the outer plexiform layer and a region corresponding to the apical processes of Müller cells. No immunofluorescence signal was detected in *Rbp1*^*−/−*^ mice on the *Abca4*^*−/−*^*/Rdh8*^*−/−*^ genetic background. In addition, the absence of the *Rbp1* transcript in the ocular tissue isolated from *Abca4*^*−/−*^*/Rdh8*^*−/−*^*/Rbp1*^*−/−*^ mice was confirmed by quantitative reverse transcription-polymerase chain reaction (qRT-PCR) (Fig. S2).

The *Abca4*^*−/−*^*/Rdh8*^*−/−*^ mice are characterized by delayed clearance of at-RAL upon light exposure and consequently are susceptible to light-induced retinal damage. Moreover, they accumulate large amounts of A2E over time, with significant buildup observed in older animals compared to WT counterparts (30). Thus, they represent a valuable model to validate potential therapeutic targets. Importantly, neither *Abca4*^*−/−*^*/Rdh8*^*−/−*^ nor *Abca4*^*−/−*^*/Rdh8*^*−/−*^*/Rbp1*^*−/−*^ mice revealed spontaneous retinal degeneration without specific exposure to intense bright light (Fig. 1*E*). Also, the visual pigment levels in dark-adapted retinas were identical in mouse lines as quantified by the amount of 11c-RAL (Fig. 1*F*). However, the absence of RBP1 in *Abca4*^*−/−*^*/Rdh8*^*−/−*^*/Rbp1*^*−/−*^ mice resulted in a slower rate of visual chromophore regeneration after exposure to bright light compared to *Abca4*^*−/−*^*/Rdh8*^*−/−*^, confirming the previously published characterization of *Rbp1*^*−/−*^ mice (25) (Fig. 1*G*). Notably, the combined absence of RDH8 and ABCA4 had a quantitatively smaller effect compared to the loss of RBP1.

**Figure 2:**
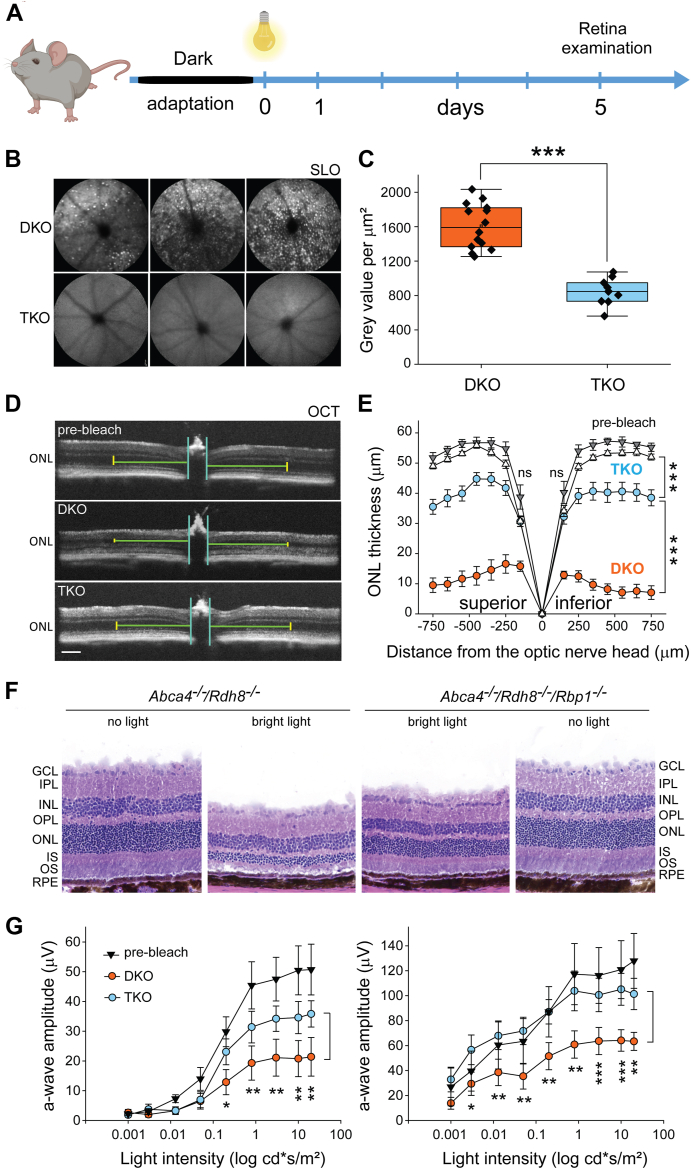
**Ocular phenotype of *Rbp1*^*−/−*^ mice on the *Abca4*^*−/−*^*/Rdh8*^*−/−*^ genetic backgrounds.***A*, schematic representation of the experimental design. The light intensity and exposure duration were chosen to induce ∼80% reduction of the retinal ONL in *Abca4*^*−/−*^*/Rdh8*^*−/−*^ mice. *B*, representative SLO images of retinas of 4-month-old mice after bright light exposure. Increased autofluorescence with a characteristic punctate pattern indicates immune cell infiltration into the damaged retina of *Abca4*^*−/−*^*/Rdh8*^*−/−*^ (DKO) mice, a feature not observed in *Abca4*^*−/−*^*/Rdh8*^*−/−*^*/Rbp1*^*−/−*^ (TKO) mice. *C*, quantification of the SLO signal shown in *panel* (*B*) reveals a statistically significant difference (∗∗∗*p* < 0.0005, unpaired *t* test) between DKO and TKO mice (9 ≤ n ≤ 14). *D*, retinal integrity assessment in light-exposed mice using OCT. ONL thickness was measured to quantify the degree of retinal degeneration. The scale bar represents 50 μm. *E*, quantification of retinal morphology changes based on OCT images. The data indicate partial protection against light-induced damage in RBP1-deficient mice (*Abca4*^*−/−*^*/Rdh8*^*−/−*^*/Rbp1*^*−/−*^ labeled as TKO) compared to their *Abca4*^*−/−*^*/Rdh8*^*−/−*^ (DKO) control counterparts. The prebleach data for DKO and TKO mice are depicted in *gray* and *white* triangles, respectively. Data represent mean ± S.D. (12 ≤ n ≤ 16). *Asterisks* indicate statistical significance between individual points (∗∗∗*p* < 0.0005, unpaired *t-*test). *F*, representative histology images corresponding to the OCT data shown in panel (*D*) confirm the protective effect of the *Rbp1* gene deletion. *G*, ERG-based functional assessment of retinal responses after exposure to damaging bright light. While *Abca4*^*−/−*^*/Rdh8*^*−/−*^ mice exhibited diminished light responses, retinal function was largely preserved in *Abca4*^*−/−*^*/Rdh8*^*−/−*^*/Rbp1*^*−/−*^ (TKO) mice (∗*p* < 0.05; ∗∗*p* < 0.005; ∗∗∗*p* < 0.0005, unpaired *t* test for two-group comparisons at individual experimental points). The prebleach data represent combined ERG responses from both mouse lines. Data represent mean ± S.D. (n = 6). ERG, electroretinogram; OCT, optical coherence tomography; ONL, outer nuclear layer; RBP1, cellular retinol-binding protein 1; SLO, scanning laser ophthalmoscopy.

To examine whether the absence of functional RBP1 affects acute retinal degeneration caused by exposure to bright light, *Abca4*^*−/−*^*/Rdh8*^*−/−*^ and background-matched *Abca4*^*−/−*^*/Rdh8*^*−/−*^*/Rbp1*^*−/−*^ mice were paired and exposed to 50,000 lux of LED light for 25 min. Five days post light exposure, fundus images were collected using scanning laser ophthalmoscopy (SLO) and optical coherence tomography (OCT) (Fig. 2*A*). *Abca4*^*−/−*^*/Rdh8*^*−/−*^ mice exhibited fundus hyperautofluorescence with a characteristic punctate pattern, indicative of infiltration of affected retinas with immune cells (Fig. 2*B*) (31). In contrast, the retinas of the corresponding RBP1-deficient *Abca4*^*−/−*^*/Rdh8*^*−/−*^*/Rbp1*^*−/−*^ mice did not display signs of increased autofluorescence and the overall autofluorescence intensity remained significantly lower as quantified in Figure 2*C*. Retinal damage in *Abca4*^*−/−*^*/Rdh8*^*−/−*^ mice was evident from a reduced retinal thickness and a diminished number of nuclei in the outer nuclear layer (ONL) as observed through OCT and histological imaging (Fig. 2, *D–F*). The quantification of ONL thickness indicated that photoreceptors in *Abca4*^*−/−*^*/Rdh8*^*−/−*^ mice were uniformly damaged across all retinal sectors (Fig. 2*E*). Notably, no significant geographic differences were observed in the preservation of retinal tissue in *Abca4*^*−/−*^*/Rdh8*^*−/−*^*/Rbp1*^*−/−*^ mice. The OCT data were confirmed by histological examination of the imaged retinas, showing largely preserved retinal morphology in *Abca4*^*−/−*^*/Rdh8*^*−/−*^*/Rbp1*^*−/−*^ mice compared to *Abca4*^*−/−*^*/Rdh8*^*−/−*^ animals (Fig. 2*F*). This reduced degeneration was also reflected in the partial preservation of light sensitivity of *Abca4*^*−/−*^*/Rdh8*^*−/−*^*/Rbp1*^*−/−*^ retinas as evidenced by significantly higher a- and b-wave amplitudes in electroretinogram (ERG) recordings (Fig. 2*G*).

**Figure 3:**
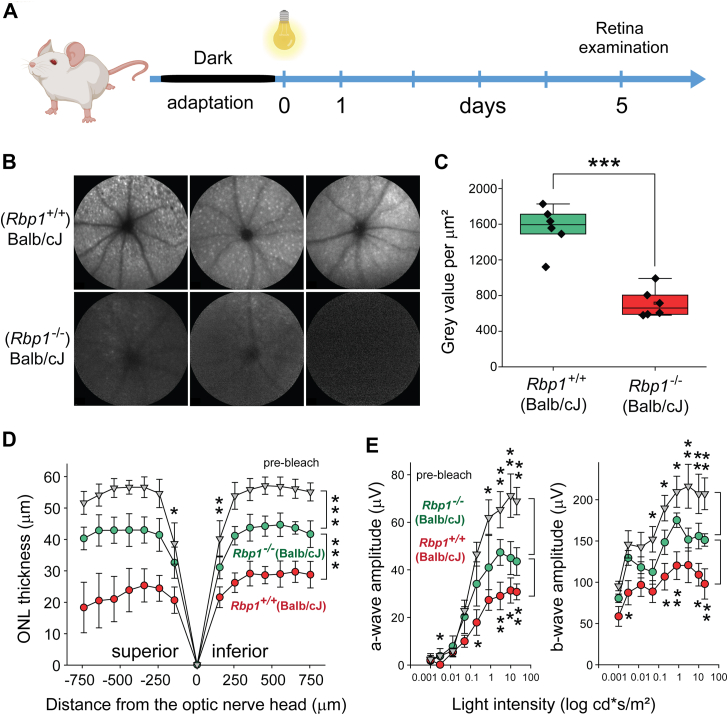
**Deletion of the Rbp1 gene decreases light sensitivity of Balb/cJ albino mice.***A*, schematic representation of the experimental design. Balb/cJ mice were exposed to bright light for 55 min, resulting in ∼50% reduction in ONL thickness. *B*, SLO images of retinas from 4-month-old albino mice after bright light exposure. Increased autofluorescence was readily observed in *Rbp1^+^*^*/^+^*^ (Balb/cJ) mice but was greatly diminished in *Rbp1*^*−/−*^ (Balb/cJ) mice, indicating a decreased in immune cell infiltration. *C*, quantification of the SLO signal revealed a statistically significant difference between *Rbp1*^*+/+*^ (Balb/cJ) and *Rbp1*^*−/−*^ (Balb/cJ) mice (∗∗∗*p* < 0.0005, unpaired *t* test, n = 6). *D*, quantification of ONL thickness in light-exposed mice. The absence of RBP1 in the light-sensitive albino mice led to partial preservation of retinal morphology (∗*p* < 0.05; ∗∗*p* < 0.005; ∗∗∗*p* < 0.0005, unpaired *t* test for two-group comparisons for individual experimental points). The prebleach data represent combined ONL thickness from both *Rbp1*^*+/+*^ (Balb/cJ) and *Rbp1*^*−/−*^ (Balb/cJ) mouse lines. The data represent mean ± SD (n = 10). *E*, preservation of retinal morphology in *Rbp1*^*−/−*^ mice, as shown in panel (*D*), correlates with sustained retinal function, particularly at higher light intensities, as assessed by scotopic ERG responses (∗*p* < 0.05; ∗∗*p* < 0.005; ∗∗∗*p* < 0.0005, unpaired *t-*test for individual experimental points). Data represent mean ± S.D. (n = 6). ERG, electroretinogram; ONL, outer nuclear layer; RBP1, cellular retinol-binding protein 1; SLO, scanning laser ophthalmoscopy.

As an alternative model of light-sensitive mice, we used the albino Balb/cJ strain to assess the role of the *Rbp1* gene deletion (32). The light sensitivity of Balb/cJ mice arises from a combination of genetic, biochemical, and anatomical factors, including RPE65 protein expression levels (12, 33), rhodopsin recycling and regeneration rates (34), and the lack of melanin pigment (35). Therefore, the generation of *Rbp1*^*−/−*^ mice on a proper Balb/cJ genetic background was critical to accurately assess the putative protective role of RBP1 deletion against light-induced retinal damage. For this purpose, we created congenic strains of *Rbp1*^*−/−*^ and *Rbp1*^+/+^ mice on Balb/cJ background by repeated backcrossing of the donor strain to the recipient strain, followed by sister-brother interbreeding of the backcrossed progeny. Starting from the sixth generation, the *Rbp1*^+/−^ progeny were tested for their light sensitivity along with Balb/cJ mice by exposing them to retinal-damaging bright light. After 10 generations, the mutant colony was maintained by breeding heterozygous mice. The resulting homozygous mutant mice (*Rbp1*^*−/−*^) were used as the KO, while WT (*Rbp1*^*+/+*^) mice served as controls. To examine the role of RBP1 ablation, these mice of nearly identical genetic backgrounds were compared for the effect of bright light on retinal integrity. Both types of mice were paired and exposed to 50,000 lux of LED light for 55 min. The retina condition was examined 5 days post light exposure, revealing a statistically significant difference in retinal autofluorescence between *Rbp*^*+/+*^ (Balb/cJ) and *Rbp1*^*−/−*^ (Balb/cJ) mice (Fig. 3, *A–C*). The reduced autofluorescence observed in RBP1-deficient animals indicates decreased infiltration of activated microglia/macrophages in response to bright light exposure, and is consistent with reduced retinal damage, as evidenced by preservation of ONL thickness (Fig. 3*D*). In addition, the observed morphological differences were associated with the partial preservation of ERG amplitudes in RBP1-deficient animals (Fig. 3*E*). Thus, the absence of functional RBP1 confers protection against light-induced retinal degeneration in two independent light-sensitive mouse models.

### The absence of RBP1 decreases the rate of A2E accumulation

**Figure 4:**
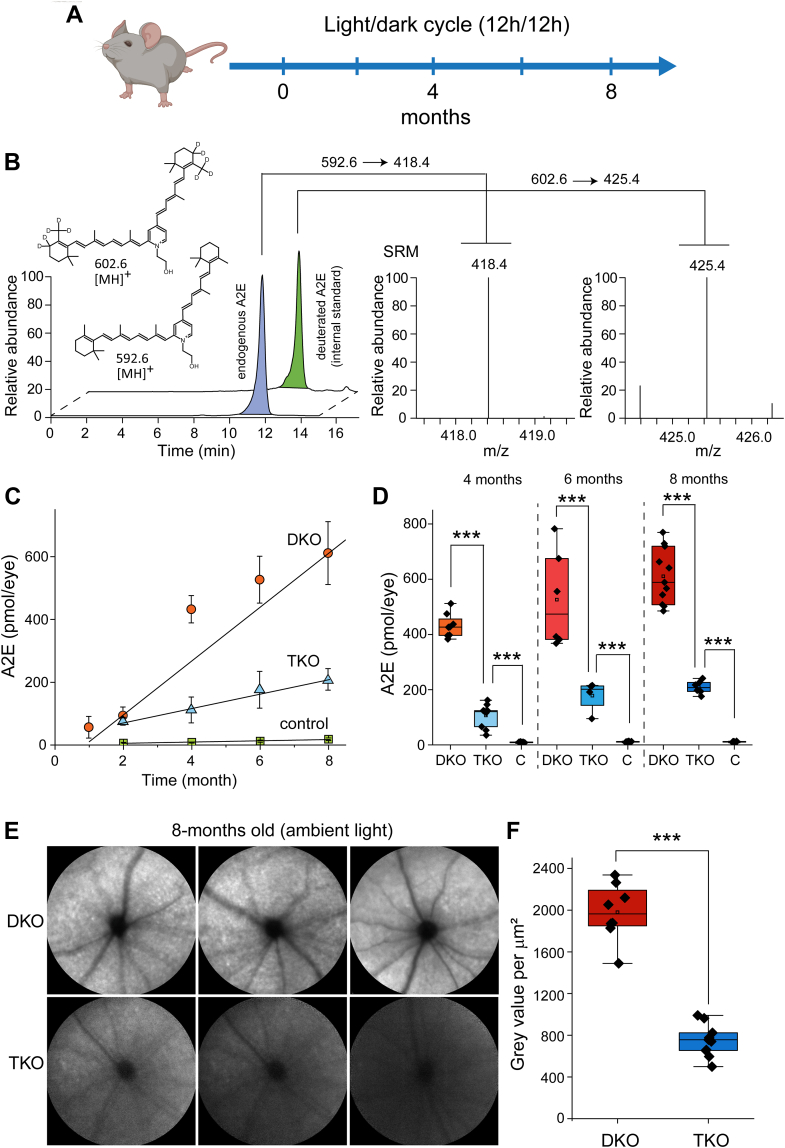
**Temporal accumulation of A2E in mouse eyes.***A*, experimental design. *Abca4*^*−/−*^*/Rdh8*^*−/−*^ (DKO), *Abca4*^*−/−*^*/Rdh8*^*−/−*^*/Rbp1*^*−/−*^ (TKO), and corresponding WT mice were maintained under a 12 h/12 h light/dark cycle for up to 8 months to assess the rate of A2E accumulation. *B*, LC/MS-based detection and quantification of A2E. Synthetic A2E-d10 was used as an internal standard to determine endogenous A2E levels. Single reaction monitoring mode was employed for the unambiguous identification of endogenous and synthetic A2E molecules in eye extracts. *C*, time-dependent increase of A2E levels in DKO, TKO, and WT mouse eyes. The calculated rate of A2E accumulation was approximately three times slower in *Abca4*^*−/−*^*/Rdh8*^*−/−*^*/Rbp1*^*−/−*^ (TKO) mice compared to *Abca4*^*−/−*^*/Rdh8*^*−/−*^ (DKO) controls. Data represent mean ± S.D. (4 ≤ n ≤ 7). *D*, Box-and-whisker plot illustrating the distribution of experimental data for samples collected from 4-month-old and 8-month-old mice, presented in *panel* (*C*) (∗∗∗*p* < 0.0005, unpaired *t-*test for two-group comparisons). *E*, representative SLO images of 8-month-old mouse retinas, showing differences in autofluorescence. The increased SLO signal across the retina correlates with higher A2E accumulation in *Abca4*^*−/−*^*/Rdh8*^*−/−*^ (DKO) mice compared to *Abca4*^*−/−*^*/Rdh8*^*−/−*^*/Rbp1*^*−/−*^ (TKO) animals. *F*, the quantification of the SLO signal is shown in *panel* (*E*) (n = 8, ∗∗∗*p* < 0.0005, unpaired *t* test for two-group comparisons). A2E, *N*-retinylidene-*N*-retinylethanolamine; LC/MS, liquid chromatography/mass spectrometry; RBP1, cellular retinol-binding protein 1; SLO, scanning laser ophthalmoscopy.

In a long-term study, we compared the accumulation of A2E in the retinas of *Abca4*^*−/−*^*/Rdh8*^*−/−*^, *Abca4*^*−/−*^*/Rdh8*^*−/−*^*/Rbp1*^*−/−*^, and WT mice used as a negative control. For this purpose, we kept both types of mice along with the corresponding control animals in ambient light conditions (∼50 lux, 12/12 h cycle) for over 8 months. Mice's eyeballs were collected at different time points (1–8 months of age), extracted with acetonitrile, and the A2E content was quantified by liquid chromatography/mass spectrometry (LC/MS) (Fig. 4, *A* and *B*). As previously shown, *Abca4*^*−/−*^*/Rdh8*^*−/−*^ mice accumulated large amounts of A2E in a time-dependent manner compared to the WT animals (30, 36) (Fig. 4, *C* and *D*). The progression of the accumulation followed a linear trend over the first 8 months, with a calculated rate of approximately 75 pmol/month. Notably, the amount of A2E found in mice lacking RBP1 was significantly smaller in those older than 2 months. This difference resulted from a three-fold slower rate of A2E accumulation in *Abca4*^*−/−*^*/Rdh8*^*−/−*^*/Rbp1*^*−/−*^ mice (∼25 pmol/month) compared to *Abca4*^*−/−*^*/Rdh8*^*−/−*^ mice (Fig. 4*C*). The increased concentration of A2E was associated with progressively higher autofluorescence in *Abca4*^*−/−*^*/Rdh8*^*−/−*^ retinas compared to *Abca4*^*−/−*^*/Rdh8*^*−/−*^*/Rbp1*^*−/−*^ mice, as observed through SLO imaging (Fig. 4, *E* and *F*). Therefore, inactivation of the *Rbp1* gene protects against the overaccumulation of A2E in this mouse model of STGD1.

### Pharmaceutical targeting of RBP1 alleviates retinal degeneration in *Abca4*^*−/−*^/*Rdh8*^*−/−*^ mice

**Figure 5:**
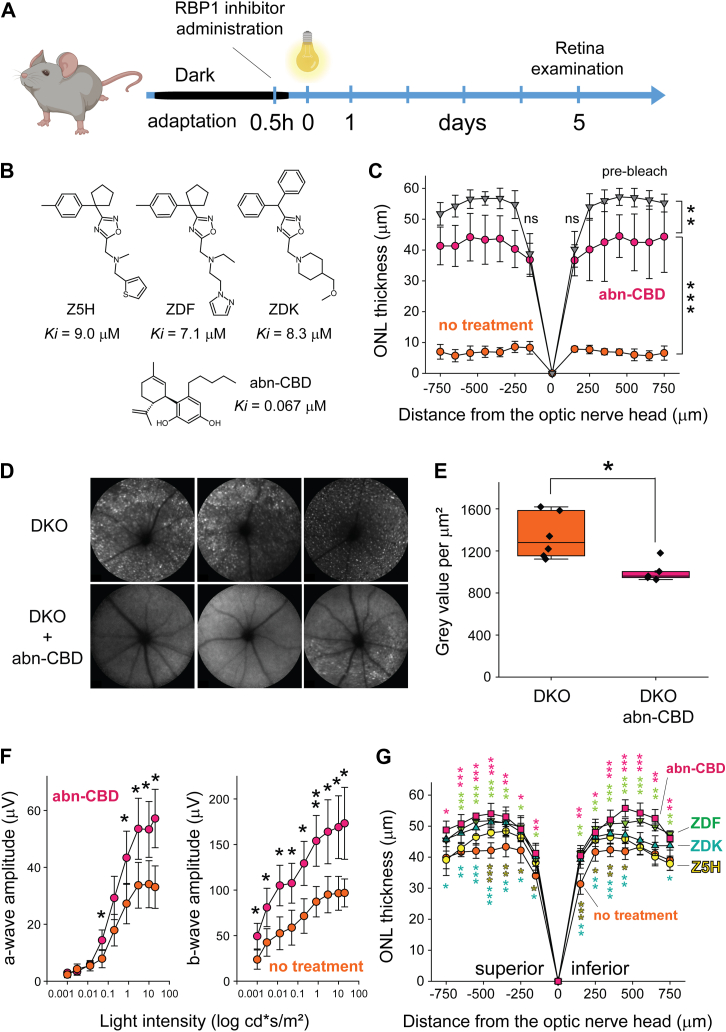
**Inhibitors of RBP1 protect against acute light-induced retinal degeneration in *Abca4*^*−/−*^*/Rdh8*^*−/−*^ mice.***A*, experimental design. RBP1 inhibitors were administered intraperitoneally to dark-adapted mice 30 min before exposure to retinal-damaging light. *B*, chemical structures of the inhibitors used in this study. Apparent *K*_*i*_ values highlight the difference in potency between abn-CBD and oxadiazol-derived compounds. *C*, effect of light exposure on retinal integrity, measured by ONL thickness. Pretreatment with abn-CBD, a potent RBP1 inhibitor, provided robust protection against acute retinal degeneration (∗∗∗*p* < 0.0005, unpaired *t* test for two-group comparisons at each experimental point). Data represent mean ± S.D. *D*, retinal autofluorescence after light exposure, assessed by SLO in abn-CBD-treated and untreated (control) mice. White puncta in the untreated group indicate inflammatory changes in the retina. *E*, quantification of SLO brightness from *panel* (*D*), showing a statistically significant difference between treated and untreated groups (∗*p* < 0.05, unpaired *t-* test, n = 6). *F*, scotopic ERG recordings reveal statistically significant preservation of retinal function in mice pretreated with abn-CBD (∗*p* < 0.05, unpaired *t* test, n = 6). abn-CBD demonstrated efficacy under high-intensity light exposure, which caused ∼80% photoreceptor degeneration in untreated mice. Data represent mean ± S.D. *G*, comparison of the protective effects of alternative RBP1 inhibitors. The efficacy of the less potent inhibitors (1–3, *panelB*) was evident only under conditions of reduced light exposure (ONL thickness reduction of ∼30%). Under these conditions, *post hoc* analysis showed significant differences between untreated animals and those injected with RBP1 inhibitors. Among the alternatives, inhibitor two exhibited the most profound retinal protective effect, based on statistical analysis (∗*p* < 0.05; ∗∗*p* < 0.005; ∗∗∗*p* < 0.0005, unpaired *t* test for individual experimental points). Data represent mean ± S.D. (n = 4). abn-CBD, abnormal cannabidiol; ERG, electroretinogram; ONL, outer nuclear layer; RBP1, cellular retinol-binding protein 1; SLO, scanning laser ophthalmoscopy.

The genetic validation of RBP1 as a biological target for the treatment of retinal diseases associated with diminished clearance of at-RAL underscores the potential therapeutic benefits for specific inhibitors of this protein. Recently, first-in-class drug candidates targeting RBP1 have been identified through high-throughput screening (27, 28). To evaluate their effectiveness in protecting against at-RAL phototoxicity, we tested selected inhibitors, including abn-CBD, Z5H, ZDF, and ZDK (Table S1) in *Abca4*^*−/−*^*/Rdh8*^*−/−*^ mice. These compounds were administered in a single dose of 30 mg/kg in dimethyl sulfoxide (DMSO) 30 min before exposing the mice to retinal-damaging light (50,000 lux, 35 min) (Fig. 5, *A* and *B*). Control mice were treated with the vehicle. The condition of the retinas was assessed *in vivo* 5 days later using SLO, OCT, and ERG recordings.

The tested compounds exhibit varying *K*_*i*_ values for RBP1, reflecting differences in their binding affinities. Among them, abn-CBD demonstrated the highest affinity, with a *K*_*i*_ of 65 nM, while compounds Z5H, ZDF, and ZDK exhibited significantly lower affinities, with *K*_*i*_ values ranging from 7 to 9 μM (27, 28). Pretreatment with abn-CBD provided substantial preservation of retinal morphology in *Abca4*^*−/−*^*/Rdh8*^*−/−*^ mice exposed to intense bright light, a condition that caused near-total retinal degeneration in the DMSO-treated control group, as measured by changes in the ONL thickness (Fig. 5*C*). abn-CBD also inhibited the post-light exposure increase in the autofluorescence of the retina, indicative of diminished infiltration of the damaged retina by immune cells (Fig. 5, *D* and *E*). The preservation of retinal morphology showed functional significance by maintaining robust photopic ERG signals as compared to untreated animals (Fig. 5*F*).

Because off-target effects may contribute to the protective action of abn-CBD, we employed the Rbp1-deficient model to assess the specificity of its mechanism of action. For this purpose, *Abca4*^*−/−*^*/Rdh8*^*−/−*^*/Rbp1*^*−/−*^ mice were treated with either abn-CBD (30 mg/kg) or vehicle (DMSO) and subjected to light-induced retinal stress, alongside a control group of *Abca4*^*−/−*^*/Rdh8*^*−/−*^ mice, which served as a control for retinal damage. Retinal morphology was evaluated by OCT 5 days post-exposure. The results showed that abn-CBD treatment did not confer additional protection in the absence of RBP1, indicating that its protective effect was specifically mediated through interaction with RBP1 rather than through alternative biological targets (Fig. S3).

Compounds characterized with lower affinity to RBP1, including Z5H, ZDF, and ZDK, exhibited no or minimal efficacy under the experimental light intensity used to validate the efficacy of abn-CBD. However, a partial protective effect was observed when exposure to bright light was lowered to the level resulting in only partial degeneration in untreated mice (50,000 lux for 20 min). Under these less severe retina-damaging conditions, inhibitors Z5H, ZDF, and ZDK demonstrated statistically significant protection against retinal damage compared to vehicle-treated animals (Fig. 5*G*). The overall protective effects of RBP1 inhibitors were influenced by light exposure conditions. Thus, as expected, differences in RBP1 affinity to their inhibitors correlated with the compounds' potencies *in vivo*. Consequently, further optimization of the pharmacodynamic properties of compounds Z5H, ZDF, and ZDK represents a promising strategy to enhance their protective effects. Overall, the above findings suggest that modulating ocular retinoid metabolism through RBP1 inhibition can mitigate the pathophysiological changes associated with light-induced photoreceptor overstimulation and at-RAL toxicity.

## Discussion

Targeting the visual cycle represents a rational, mechanism-based therapeutic strategy for retinal degenerative diseases, offering hope for preserving vision and improving the quality of life for affected patients. By addressing the underlying biochemical pathway involved in retinal dysfunction, visual cycle modulators have the potential to transform the treatment paradigm for retinopathies caused by insufficient clearance of at-RAL, including STGD1, certain subtypes of rod-cone dystrophies, and other related conditions. Various strategies and potential biological targets have been proposed to control retinoid metabolism in the eye, such as direct inhibition of RPE65 and RBP4, or chemical mitigation of at-RAL reactivity through the isotopic effect of deuterated retinoids, or sequestration in the form of stable Schiff-base (19, 37, 38, 39). These approaches provided initial proof that the rate of visual chromophore regeneration and bis-retinoid formation can be influenced pharmacologically. However, some of the clinical trials have shown that these strategies might not represent the safest or most effective therapeutic options.

In this context, identifying alternative, more applicable biological targets to control retinoid metabolism in the eye appears to be the next logical step in developing visual cycle modulators suitable for clinical use. The recent discovery of nonretinoid inhibitors of RBP1 provided chemical tools to influence the activity of this protein *in vivo* (27, 28). However, until now, there has been no evidence supporting the consequential role of RBP1 in preventing retinopathies, hindering further development of therapies targeting this protein. Therefore, we investigated the effects of inactivating the *Rbp1* gene on retinal health in both acute and long-term experimental settings. In agreement with the initial findings by Saari *et al.* (25), the absence of RBP1 contributed to a moderate decrease in the rate of visual cycle regeneration regardless of the genetic background of the examined mice. RBP1 plays an important role in driving the diffusion of at-ROL from the neural retina to the RPE cells, attributable to its ability to deliver at-ROL to lecithin:retinol acyltransferase (LRAT), the main retinol esterification enzyme in the RPE cells (40, 41, 42).

**Figure 6:**
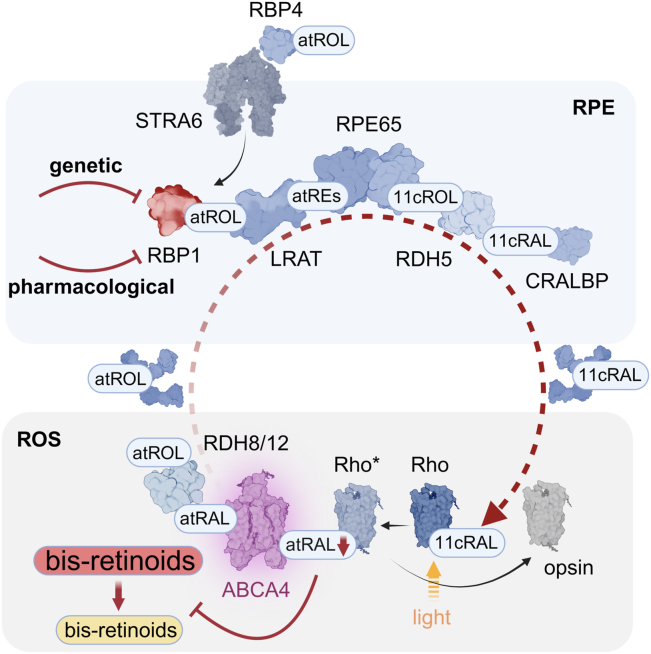
**The role of RBP1 in retinoid homeostasis and the mechanistic basis for the protective effect of its inhibitors.** RBP1 facilitates the intracellular transport of at-ROL in RPE cells. Its absence delays the regeneration of the visual chromophore by slowing down at-ROL esterification by LRAT, leading to a transient accumulation of at-ROL compared to WT mice. An unforeseen consequence of RBP1 deficiency is diminished light sensitivity in mice susceptible to light-induced retinal damage, along with a slower accumulation of at-RAL-derived side metabolites. Pharmacological inhibition of RBP1 mimics the phenotype of *Rbp1*^*−/−*^ mice, offering a potential therapeutic strategy for modulating the visual cycle. Therefore, RBP1 inhibitors may provide an alternative approach to treating diseases associated with impaired clearance of at-RAL and excessive bis-retinoid accumulation. LRAT, lecithin:retinol acyltransferase; RBP1, cellular retinol-binding protein 1; RPE, retinal pigment epithelium.

However, it was unclear whether the relatively moderate effect of *Rbp1* deletion on the visual cycle would be sufficient to overcome pathological changes in mouse models of retinopathies. The data presented in this manuscript provide compelling evidence for the protective role of *Rbp1* inactivation against light-induced retinal degeneration and the long-term overaccumulation of A2E in a mouse model of STGD1 (Fig. 6). The observed phenotype of *Rbp1*^*−/−*^ mice in acute retinal damage experiments can largely be attributed to averting overstimulation of photoreceptor cells and the transient excess of at-RAL (36).

Prolonged inhibition of the visual cycle leads to a decreased steady-state concentration of at-RAL, thereby lowering the rate of A2E formation. Importantly, our data suggest that incomplete inhibition of the visual cycle through the absence of RBP1 can have a significant therapeutic effect. This finding contrasts with previous data obtained for RPE65, in which nearly complete and prolonged inhibition was required to achieve retinal protection or decrease A2E accumulation in *Abca4*^*−/−*^*/Rdh8*^*−/−*^ mice (30, 43). A similar observation was true for mice carrying the RPE65 Leu450/Met variant, where protection against light-dependent retinal damage occurred only in homozygous mice with a 5-fold slower rate of rhodopsin regeneration (12).

Our findings indicate that RBP1 primarily facilitates retinoid transport and enhances the rate of retinoid biotransformation, rather than directly enabling visual chromophore regeneration. Although chromophore regeneration still occurs in *Rbp1*^*−/−*^ mice, it does so with reduced efficiency, highlighting the modulatory role of this protein in the visual cycle. This functional nuance is critical in interpreting the partial protection against light-induced retinal damage observed at the 30 mg/kg abn-CBD dose in WT albino mice (27). Although initially it was unclear whether this outcome reflected suboptimal target saturation with the inhibitor or the intrinsic limitations of inhibiting RBP1, data from *Rbp1*^*−/−*^ mice now support the latter interpretation. These results underscore that pharmacological inhibition of RBP1 can only partially suppress the visual cycle, consistent with its physiological role in supporting, but not performing, key steps in chromophore regeneration.

RBP1 is the only validated biological target within the visual cycle whose genetic deletion does not cause spontaneous retinal degeneration. In contrast, based on animal studies and human data, the inactivation of any other tested or proposed proteins, such as RPE65, LRAT, RBP4, or CRALBP, is associated with potential adverse side effects affecting the health of photoreceptors (44, 45, 46, 47). Thus, targeting RBP1 might offer a safer alternative to previously studied therapeutic strategies (Fig. 6). However, this favorable safety profile comes at the cost of reduced efficacy in preventing light-induced retinal damage and less robust inhibition of A2E accumulation when compared to RPE65 inhibitors like emixustat and its derivatives. Moreover, the main systemic disadvantage of *Rbp1* deletion is a reduction in hepatic retinyl esters and an increased turnover of retinoids in the liver, which can lead to higher susceptibility to vitamin A deficiency (26). However, this phenotype is diet-dependent, and maintaining a vitamin A-sufficient diet should be adequate to mitigate the adverse effects associated with the inhibition of RBP1.

Although less abundant than in the RPE, RBP1 is detectable in the apical processes of Müller glial cells (Fig. S1), suggesting a potential role for this protein in the photic regeneration of cone visual pigments. However, the functional significance of RBP1 in Müller cells remains unclear at this time. A direct approach to investigate this would involve generating *Rbp1*^*−/−*^*/Gnat1*^*−/−*^ double KO mice and analyzing cone pigment regeneration kinetics under photopic conditions. Nevertheless, pharmacological inhibition of RBP1 may exert additional effects on the visual system beyond the canonical visual cycle, which warrants careful evaluation in cone-dominant animal models.

Due to their critical roles in trafficking and signaling, lipid-binding carriers, particularly fatty acid-binding proteins, have been extensively investigated as potential therapeutic targets for metabolic, inflammatory, neurological, and cancerous diseases (48, 49). However, several challenges, such as functional redundancy, broad biological roles, lack of specificity, compensatory mechanisms, and off-target effects, have hindered the development of drugs targeting these proteins. Another difficulty with targeting lipid-binding proteins is the need for their saturation with a drug to achieve a therapeutic effect, implying relatively high concentrations *in vivo*. These obstacles have contributed to the lack of Food and Drug Administration-approved lipid-binding-targeting drugs. Nevertheless, the specific role of RBP1 in ocular physiology and promising preclinical data may suggest that inhibitors of this protein could be the first-in-class drug candidates among lipid-transporting proteins.

As research continues to elucidate the complexities of the visual cycle and its role in retinal degeneration, novel therapeutic approaches and combination therapies may meaningfully enhance treatment outcomes and improve the prognosis for patients with debilitating retinal degeneration. New clinical trials and technological advancements are essential to realizing the full therapeutic potential of visual cycle modulators and advancing the field of retinal medicine in the quest to combat vision loss.

## Experimental procedures

### Chemicals and reagents

4-[(1R,6R)-3-methyl-6-prop-1-en-2-ylcyclohex-2-en-1-yl]-5-pentylbenzene-1,3-diol (abnormal cannabidiol abbreviated as abn-CBD) from Cayman Chemical. The remaining compounds tested in this study, including N-methyl-N-[[3-[1-(4-methylphenyl)cyclopentyl]-1,2,4-oxadiazol-5-yl]methyl]-1-thiophen-2-ylmethanamine (Z5H), N-ethyl-N-[[3-[1-(4-methylphenyl)cyclopentyl]-1,2,4-oxadiazol-5-yl]methyl]-2-pyrazol-1-ylethanamine (ZDF), and 3-benzhydryl-5-[[4-(methoxymethyl)piperidin-1-yl]methyl]-1,2,4-oxadiazole (ZDK) were purchased from Hit2Lead (Table S1). Deuterated at-RAL (at-RAL-5d) was purchased from LGC Standards and served as a substrate to synthesize deuterated *N*-retinylidene-*N*-retinylethanolamine (A2E-10d) using a previously published method (50). In the final step, A2E-10d was purified by reverse phase chromatography using a Gemini 5 μm C18 column 250 × 4.6 mm (Phenomenex) in a gradient to acetonitrile/isopropanol (50/50 v/v) in water.

### Animal breeding and care

All animal procedures and experiments were approved by the Case Western Reserve University Animal Care Committees and conformed to both the recommendations of the American Veterinary Medical Association Panel on Euthanasia and the Association for Research in Vision and Ophthalmology. All animals were housed and bred in the CWRU Animal Resource Center of the School of Medicine, under a 12-h light (ambient illuminance ∼50 lux) and 12-h dark cycle. Mice were maintained on a Prolab RMH 3000 rodent diet (LabDiet), containing 18 IU/g of vitamin A. All animal procedures and experimental protocols were approved by the Institutional Animal Care and Use Committee at CWRU and adhered to the recommendations of the American Veterinary Medical Association Panel on Euthanasia and the Association for Research in Vision and Ophthalmology. *Abca4*^*−/−*^/*Rdh8*^*−/−*^ mice (Strain #: 030503) were purchased from the Jackson Laboratory. Upon receipt, genetic homogeneity and preservation of the essential genetic background were ensured through selective breeding and genotyping, with special attention to the RPE65 Leu450/Met variant and *rd8* mutation. *Rbp1*^*−/−*^ mice were generously provided by Dr W.S. Blaner from Columbia University, New York, NY. Upon receipt, these mice underwent rederivation using C57BL/6J WT females as donors to obtain heterozygous offspring. These heterozygous mice were subsequently bred for multiple generations to reestablish the *Rbp1*^*−/−*^ mouse line.

To generate experimental mice for evaluating the role of RBP1 on the *Abca4*^*−/−*^*/Rdh8*^*−/−*^ background, we initially crossed *Abca4*^*−/−*^*/Rdh8*^*−/−*^ mice with *Rbp1*^*−/−*^ mice. The resulting F1 progeny were heterozygous at all three loci (*Abca4*^*+/−*^*/Rdh8*^*+/−*^*/Rbp1*^*+/−*^) and were intercrossed to produce F2 and subsequent generations. Through selective breeding and genotyping, we obtained mice with various genotypes, including double knockouts (*Abca4*^*−/−*^*/Rdh8*^*−/−*^*/Rbp1*^*+/+*^) and triple knockouts (*Abca4*^*−/−*^*/Rdh8*^*−/−*^*/Rbp1*^*−/−*^). This breeding strategy allowed us to use littermates or closely related siblings for experimental comparisons, ensuring consistent genetic backgrounds across groups.

A key challenge in generating the *Abca4*^*−/−*^*/Rdh8*^*−/−*^*/Rbp1*^*−/−*^ triple knockout (TKO) line was the genetic linkage between *Rdh8* and *Rbp1*, both located on chromosome 9, but at different genetic positions: *Rbp1* at 51.36 centimorgans (cM) and *Rdh8* at 7.64 cM, corresponding to a genetic distance of approximately 43.72 cM. This indicates a moderate probability of recombination between these two loci during meiosis (with 1 cM ≈ 1% recombination frequency). To more accurately estimate the recombination frequency (r) between *Rbp1* and *Rdh8*, we applied the Haldane mapping function:

$\text{mfc}=1/4\left\{1/2\left[\ln\left(1+2r\right)-\ln\left(1-2r\right)\right]+\tan^{-1}\left(2r\right)\right\}\text{,}$ (51)

Where mfc represents genetic distance in Morgans (mfc = 0.4372 M). The resulting calculated r value is 0.3963, corresponding to a ∼39.6% chance that a recombination event will occur between the *Rbp1* and *Rdh8* loci in a single meiosis. Since recombination frequency can approach, but not exceed, 50%, this relatively high value indicates that it was possible to generate the *Abca4*^*−/−*^*/Rdh8*^*−/−*^*/Rbp1*^*−/−*^ mouse line, although with a lower probability of approximately 1/102 (∼0.98%) compared to 1/64 (∼1.56%) for unlinked genes. This calculation accounts for the need for recombination between *Rbp1* and *Rdh8* in both parental gametes and Mendelian segregation of the unlinked *Abca4* locus. Specifically, recombinant gametes occur with a probability of r/2 per parent, resulting in an overall TKO frequency of (r/2)^2^ × (1/4). This is a significantly lower probability, necessitating the screening of a large number of offspring to identify the desired TKO genotype. To partially overcome this challenge, we implemented selective breeding strategies to enrich for recombinant haplotypes over successive generations. All experimental mice were genotyped for *Abca4*, *Rdh8*, *Rbp1*, *RPE65* Leu450/Met variant, and *rd8* mutations (Fig. 1*B*).

To generate *Rbp1*^*−/−*^ mice on the albino Balb/cJ genetic background, the rederived female *Rbp1*^*−/−*^ strain was crossed with male Balb/cJ mice (strain #000651, The Jackson Laboratory). The resulting heterozygous offspring (carrying the mutation and the Balb/cJ background alleles) were backcrossed to Balb/cJ mice. Select offspring carrying the mutant allele from each generation were further bred with Balb/cJ mice. This process was repeated for 10 generations to progressively incorporate the Balb/cJ genetic background. Throughout the backcrossing process, mice were genotyped to confirm the presence of the mutant allele. After 10 generations, the mutant colony was maintained by breeding heterozygous mice. The resulting homozygous mutant mice (*Rbp1*^*−/−*^) were used as the KO, while wild-type (*Rbp1*^*+/+*^) mice served as controls.

Except for studies involving long-term ocular A2E accumulation, all mice used in the experiments were between 2 and 4 months old at the time of analysis, ensuring consistency in age-related factors that could influence experimental outcomes.

### Mouse genotyping

Mouse genotyping was performed using genomic PCR. The specific primer sequences, PCR cycling conditions, and expected amplicon sizes for each target locus are provided in Table S2. Prior to initiating the breeding scheme, we confirmed the absence of the *Rpe65* Leu450Met polymorphism and the *rd8* mutation in both the *Abca4*^*−/−*^*/Rdh8*^*−/−*^ and *Rbp1*^*−/−*^ parental lines to eliminate potential confounding background effects on retinal phenotypes. To ensure genetic consistency throughout the study, genotyping for the *Rpe65* variant and *rd8* was repeated at the conclusion of the experiments. This *post hoc* validation step confirmed the stability of the genetic background in all experimental animals. The same genotyping and validation protocol was applied to the *Rbp1*^*−/−*^ line maintained on an albino background to verify the absence of these confounding alleles in that strain as well.

### Detection and quantification of visual chromophore

The procedure was adapted from a previously published protocol (52). To extract 11cRAL, eyes were enucleated from dark-adapted, euthanized mice. Two eyes per sample were homogenized in a glass/glass homogenizer containing 1 ml of 40 mM hydroxylamine in a 1:1 PBS/methanol solution (v/v). The homogenate was incubated at room temperature for 20 min to derivatize retinyl aldehydes into their corresponding oximes. Retinoids were then extracted with 4 ml of hexane, followed by centrifugation at 5000*g* for 10 min. The upper organic phase was collected in a glass test tube, dried using a rotary SpeedVac, and redissolved in 250 μl of hexane for HPLC analysis. Retinoid separation was performed on a normal-phase column (Agilent Zorbax Sil, 5 μm, 4.6 × 250 mm) using a step gradient of ethyl acetate in hexane (1% for 10 min, then 10% for 40 min) at a constant flow rate of 1.5 ml/min (Agilent 1200 HPLC system). Elution was monitored at 325 and 360 nm. 11cRAL oximes were identified based on their retention times and characteristic UV/visible spectra, while retinoid quantification was determined by linear correlations between synthetic standard injections and chromatographic peak areas.

### A2E extraction and LC/MS-based quantification

All procedures involving eye handling and retinoid extraction were conducted under dim light conditions. To extract A2E, two frozen mouse eyes were placed into a glass test tube containing 1 ml of ice-cold acetonitrile. The eyes were then mechanically homogenized using an electrical tissue homogenizer (USA Scientific). The homogenate was enriched with 5 to 50 pmol of A2E-10d, a synthetic internal standard, and transferred to a dounce glass-glass tissue grinder for a second round of homogenization. The homogenate underwent centrifugation at 15,000*g* for 5 min, and the resulting clear supernatant was carefully collected. The organic solvent was evaporated using a SpeedVac, and the residual A2E was dissolved in 0.2 ml of acetonitrile before being transferred into an HPLC vial for LC/MS analysis.

For analysis, individual samples (100 μl) were injected onto an XBridge BEH C4 reverse phase HPLC column (3.5 μm, 2.1 mm × 50 mm, Waters). A linear gradient of acetonitrile in water, ranging from 30% to 100% (v/v), was employed for the elution of A2E. All solvents utilized contained 0.1% formic acid (v/v). The gradient was developed over 10 min at a flow rate of 0.3 ml/min. The detection and quantification of A2E were accomplished using an LTQ Velos linear ion trap mass spectrometer (Thermo Fisher Scientific) equipped with an electrospray ionization source. Optimal ionization and detection parameters were established using a synthetic A2E standard to ensure maximum sensitivity. A2E levels in the analyzed samples were quantified based on the correlation between the area under the ion intensity peak corresponding to a specific fragmentation of the endogenous A2E ion (m/z = 592.6 [MH]+ → 418.4) and the corresponding deuterated internal standard (m/z = 602.6 [MH]+ → 425.4). A2E levels are reported as pmol/eye. This study used eyes from males and females of different ages (1, 2, 4, 6, and 8 months) to analyze A2E levels. Each group consisted of four to seven mice.

### Light-induced retinal degeneration

Retinal degeneration was induced by exposing dark-adapted mice to white light with an intensity of approximately 50,000 lux. The light was emitted from two 100-W ZHMA IP66 diode lamps (Home Depot) positioned on the left and right sides of the cage, with two mice per cage per exposure set. The spectrum of emitted light was provided in Fig. S4. Before exposure, pupils were dilated with a mixture of 1% cyclopentolate HCL, 1% tropicamide, and 2.5% phenylephrine HCl (Leiters). The duration of light exposure varied depending on the experiment and genotype and was determined experimentally. Through extensive testing, we optimized conditions that consistently produced reproducible retinal damage in *Abca4*^*−/−*/^*Rdh8*^*−/−*^ mice, resulting in a ∼80% reduction of ONL thickness from 60 μm (prebleach) to ∼10 μm (5 days post bleach), as measured by OCT. Twenty-five minutes of light exposure was used for comparing the light sensitivity of DKO and TKO mice and for testing the efficacy of abn-CBD. For the partial light damage model used to evaluate less potent RBP1 inhibitors, the exposure time was reduced to 20 min. Tested inhibitors of RBP1 were administered intraperitoneally at a dose of 30 mg/kg in sterilized DMSO 30 min before bright light exposure. For *Rbp1*^*−/−*^ and *Rbp1*^*+/+*^ mice on the albino Balb/cJ genetic background, the exposure time was extended to 55 min, as they revealed lower susceptibility to the bright light retinal damage. Retinal thickness, fundus autofluorescence, and function were analyzed *in vivo* using OCT, SLO, and ERG, respectively, 5 days after light exposure. There was no statistically significant difference in the magnitude of retinal degeneration between the sexes in *Abca4*^*−/−*/^*Rdh8*^−/−^ or Balb/cJ mice (Fig. S5). Therefore, for the retinal degeneration experiments, data from both sexes were combined to calculate mean values ± SD

### Optical coherence tomography

Mice were anesthetized with an intraperitoneal injection of a mixture containing 20 mg/ml ketamine and 1.75 mg/ml xylazine in sterile saline, administered at a dose of 0.15 ml per 25 g of body weight. Before imaging, pupils were fully dilated using either 1% tropicamide (Akorn, Inc) or a mixture of 1% cyclopentolate HCL, 1% tropicamide, and 2.5% phenylephrine HCl (Leiters). OCT images were acquired in rectangular B-scan mode (1.8 × 1.8 mm, 1000 × 100 × 8 average) at 0° and 90° using a G4 mouse lens on an ultra-high-resolution OCT instrument (Envisu R2210 UHR 120V, Leica Microsystems Inc).

### Scanning laser ophthalmoscope

Mouse fundus imaging was performed with a confocal scanning laser ophthalmoscope (HRA Spectralis; Heidelberg Engineering) equipped with a 55-degree lens on an anesthetized mouse. To obtain a well-aligned and evenly illuminated fundus image, the near-infrared reflectance mode (820 nm) was used to position the fundus camera relative to the pupil. Fundus autofluorescence was monitored using 488 nm excitation with an emission filter range of 500 to 700 nm.

### Electroretinography analyses

Before ERG recording, the pupils of dark-adapted, anesthetized mice were dilated with 1% tropicamide (Akorn, Inc), and each eye was covered with hypromellose 0.3% gel (GenTeal Tears Lubricant Eye Gel, Alcon) as a conductive gel. Scotopic threshold ERGs were recorded for both eyes of each mouse using Ag/AgCl electrodes on a Celeris rodent ERG system (Diagnosys) with Diagnosys Espion V6 software.

To evaluate the differences in dark adaptation between *Abca4*^*−/−*^*/Rdh8*^*−/−*^ and *Abca4*^*−/−*^*/Rdh8*^*−/−*^*/Rbp1*^*−/−*^ mice, ERG recordings were performed 90 min after exposure to two consecutive light flashes from a Neewer TT560 photographic flash unit at maximum intensity, which resulted in the photobleaching of approximately 80% of the visual pigment.

Scotopic responses were also obtained 7 days after light-induced retinal degeneration for *Abca4*^*−/−*^*/Rdh8*^*−/−*^ and *Abca4*^*−/−*^*/Rdh8*^*−/−*^*/Rbp1*^*−/−*^ mice, *Rbp1*^*−/−*^ and *Rbp1*^*+/+*^ mice on an albino Balb/cJ genetic background, and Abca*4*^*−/−*^*/Rdh8*^*−/−*^ mice with and without RBP1 inhibitors. All the scotopic responses were recorded in darkness using nine steps of white flash luminance, ranging from 0.001 to 20 cd.s.m^−2^. The interstimulus interval increased from 7 s for low-luminance flashes to 70 s for the highest intensity stimuli.

### Retinal histology

The structural morphology of mouse retinas exposed to bright light was assessed using H&E staining of fresh-frozen tissue. Eye tissues were sectioned at 10 μm thickness using a Leica CM1950 cryostat. The sections were then dried for 2 h at 37 °C, fixed in 4% paraformaldehyde, washed with PBS, and stained with H&E. Wide-field images of H&E-stained sections were acquired using a Zeiss AxioScan.Z1 equipped with a Hitachi HV-F203 color camera and a 20×/0.8 Plan-Apochromat objective (Carl Zeiss Research Microscopy Solution). Each image was composed of multiple tiles stitched together after acquisition with the Zen Blue software.

### Immunofluorescence staining

Mouse eyes were collected and immediately fixed in 4% formaldehyde-PBS solution. Twenty-four hours later, they were placed in a 30% sucrose solution in PBS. After the tissues were fully saturated with sucrose, they were embedded in optimal cutting temperature compound medium (Sakura Fine Chemicals) and then frozen. Cryosections were cut using a cryostat microtome (Leica) at 5 μm thickness. Sections were collected onto Fisher Superfrost Plus slides and allowed to dry, then stored at −20 °C. Frozen sections were permeabilized and blocked in 2% donkey serum (Millipore Sigma), 1% bovine serum albumin (Vector Laboratories), and 0.1% Triton X-100 (Millipore Sigma) in PBS. Then, the sections were incubated overnight at 4 °C with the rabbit anti-RBP1 polyclonal antibody (Proteintech) at a 1:50 dilution or anti-glutamine synthetase polyclonal antibody (MilliporeSigma) at a 1:100 dilution to identify Müller glia cells. Next, the sections were washed three times with PBS for 10 min and incubated for 2 h with a 1:400 dilution of the donkey anti-rabbit NL637-conjugated secondary antibody (R&D Systems). The sections were washed three times for 15 min each with PBS, incubated with 1 μM Hoechst (AB228551, Abcam) for 30 min, and mounted in mounting medium (ProLong Gold Antifade Reagent, Invitrogen) and covered with cover glass, and allowed to dry. Fluorescent images were obtained on an Olympus FV1200 Laser Scanning Microscope using laser diodes 405 nm for Hoescht-stained nuclei and 647 nm for stained proteins of interest with a 40× objective.

### Western blotting of mouse eye homogenates

Two mouse eyes were homogenized in ice-cold 250 μl of 10 mM Tris–HCl buffer (pH 8.0) using a glass-glass Dounce tissue homogenizer. The homogenate was centrifuged at 15,000*g* for 15 min at 4 °C to isolate the soluble protein fraction. Twenty-five microliters of the sample was loaded onto a 4 to 20% gradient SDS-PAGE mini gel (Bio-Rad). Separated proteins were transferred to a polyvinylidene difluoride membrane (Bio-Rad). After blocking with 5% skim milk in TBST buffer (25 mM Tris–HCl, pH 7.4, 150 mM NaCl, and 0.1% Tween-20) for 1 h, the membrane was incubated for 1 h with primary anti-RBP1 rabbit polyclonal antibody (Proteintech) diluted 1:2500 in TBST and anti-actin rabbit monoclonal antibody (Cell Signaling Technology). The primary antibody was validated using eye tissues isolated from *Rbp1*^*−/−*^ mice. The membrane was washed three times with TBST and then incubated with a secondary goat anti-rabbit HRP-conjugated antibody (Promega) diluted 1:3000 in TBST. After three additional washes with 25 mM Tris–HCl (pH 7.4) and 150 mM NaCl, the membrane was developed using SuperSignal West Pico PLUS chemiluminescent substrate (Thermo Fisher Scientific). Signals related to the presence of RBP1 and actin were detected using the Odyssey XF imaging system (LI-COR Biosciences).

### qRT-PCR analyses

Whole eyes samples collected from *Abca4*^*−/−*^*/Rdh8*^*−/−*^ and *Abca4*^*−/−*^*/Rdh8*^*−/−*^*/Rbp1*^*−/−*^ mice were preserved in RNAlater solution (Sigma-Aldrich) until analysis. Total RNA was extracted using the RNeasy Mini Kit (Qiagen) according to the manufacturer’s instructions. RNA concentration and purity were assessed using a NanoDrop spectrophotometer (ND-1000, Thermo Fisher Scientific). Up to 2 μg of total RNA was reverse transcribed into complementary DNA in a 20 μl reaction using the High-Capacity cDNA Reverse Transcription Kit (Applied Biosystems). qRT-PCR was performed using TaqMan probes (Applied Biosystems) for *Rbp1* (Mm00441119_m1), with *Gapdh* (Mm99999915_g1) serving as an endogenous control. All real-time PCR experiments were conducted on an ABI StepOnePlus qRT-PCR instrument (Applied Biosystems).

### Statistical analysis

Data are presented as mean ± SD or box-and-whiskers plot graphs. The boxes represent SD values, the mean is marked by a vertical line inside the box, whereas the whiskers correspond to min and max values. The number of samples and independent experiments is indicated in the figure legends. Student’s *t-*test (unpaired, 2-tailed) was employed for two-group comparisons following the normal distribution check by the Shapiro–Wilk test. Statistical significance was considered at a *p-*value less than 0.05. The ranges of *p-*values for individual experimental groups are provided in the figure legends. The statistical calculations were performed using Origin 2024 (OriginLab) or Excel (Microsoft) software.

## Data availability

All data supporting the findings of this study are available from the corresponding author upon reasonable request. Any additional information required to reanalyze the data reported in this article is available from the lead contact upon request.

## Supporting information

This article contains supporting information.

## Conflict of interest

M. G. is a co-author of a pending patent related to the use of RBP1 inhibitors (US20210228500A1). Other authors declare that they have no conflicts of interest with the contents of this article.

## References

[1] Kiser P.D., Golczak M., Palczewski K. (2014). Chemistry of the retinoid (visual) cycle. Chem. Rev..

[2] Kiser P.D., Palczewski K. (2021). Pathways and disease-causing alterations in visual chromophore production for vertebrate vision. J. Biol. Chem..

[3] Lamb T.D. (2022). Photoreceptor physiology and evolution: cellular and molecular basis of rod and cone phototransduction. J. Physiol..

[4] Sato S., Kefalov V.J. (2024). The retina-based visual cycle. Annu. Rev. Vis. Sci..

[5] Travis G.H., Golczak M., Moise A.R., Palczewski K. (2007). Diseases caused by defects in the visual cycle: retinoids as potential therapeutic agents. Annu. Rev. Pharmacol. Toxicol..

[6] Maeda T., Golczak M., Maeda A. (2012). Retinal photodamage mediated by all-trans-retinal. Photochem. Photobiol..

[7] Zhao J., Kim H.J., Ueda K., Zhang K., Montenegro D., Dunaief J.L. (2021). A vicious cycle of bisretinoid formation and oxidation relevant to recessive Stargardt disease. J. Biol. Chem..

[8] Donato L., D'Angelo R., Alibrandi S., Rinaldi C., Sidoti A., Scimone C. (2020). Effects of A2E-Induced oxidative stress on retinal epithelial cells: new insights on differential gene response and retinal dystrophies. Antioxidants (Basel).

[9] Kotnala A., Senthilkumari S., Wu G., Stewart T.G., Curcio C.A., Halder N. (2022). Retinal pigment epithelium in human donor eyes contains higher levels of bisretinoids including A2E in periphery than macula. Invest. Ophthalmol. Vis. Sci..

[10] Piotter E., McClements M.E., MacLaren R.E. (2021). Therapy approaches for Stargardt disease. Biomolecules.

[11] Kubota R., Birch D.G., Gregory J.K., Koester J.M. (2022). Randomised study evaluating the pharmacodynamics of emixustat hydrochloride in subjects with macular atrophy secondary to Stargardt disease. Br. J. Ophthalmol..

[12] Wenzel A., Reme C.E., Williams T.P., Hafezi F., Grimm C. (2001). The Rpe65 Leu450Met variation increases retinal resistance against light-induced degeneration by slowing rhodopsin regeneration. J. Neurosci..

[13] Sieving P.A., Chaudhry P., Kondo M., Provenzano M., Wu D., Carlson T.J. (2001). Inhibition of the visual cycle in vivo by 13-cis retinoic acid protects from light damage and provides a mechanism for night blindness in isotretinoin therapy. Proc. Natl. Acad. Sci. U. S. A..

[14] Kim S.R., Fishkin N., Kong J., Nakanishi K., Allikmets R., Sparrow J.R. (2004). Rpe65 Leu450Met variant is associated with reduced levels of the retinal pigment epithelium lipofuscin fluorophores A2E and iso-A2E. Proc. Natl. Acad. Sci. U. S. A..

[15] Hussain R.M., Gregori N.Z., Ciulla T.A., Lam B.L. (2018). Pharmacotherapy of retinal disease with visual cycle modulators. Expert Opin. Pharmacother..

[16] Bavik C., Henry S.H., Zhang Y., Mitts K., McGinn T., Budzynski E. (2015). Visual cycle modulation as an approach toward preservation of retinal integrity. PLoS One.

[17] Kiser P.D., Palczewski K. (2016). Retinoids and retinal diseases. Annu. Rev. Vis. Sci..

[18] Swigris J., Widjaja-Adhi M.A.K., Golczak M. (2025). Retinoid dynamics in vision: from visual cycle biology to retina disease treatments. Pharmacol. Ther..

[19] Golczak M., Kuksa V., Maeda T., Moise A.R., Palczewski K. (2005). Positively charged retinoids are potent and selective inhibitors of the trans-cis isomerization in the retinoid (visual) cycle. Proc. Natl. Acad. Sci. U. S. A..

[20] Kiser P.D., Zhang J., Badiee M., Kinoshita J., Peachey N.S., Tochtrop G.P. (2017). Rational tuning of visual cycle modulator pharmacodynamics. J. Pharmacol. Exp. Ther..

[21] Racz B., Varadi A., Kong J., Allikmets R., Pearson P.G., Johnson G. (2018). A non-retinoid antagonist of retinol-binding protein 4 rescues phenotype in a model of Stargardt disease without inhibiting the visual cycle. J. Biol. Chem..

[22] Maeda A., Golczak M., Chen Y., Okano K., Kohno H., Shiose S. (2011). Primary amines protect against retinal degeneration in mouse models of retinopathies. Nat. Chem. Biol..

[23] Ma L., Kaufman Y., Zhang J., Washington I. (2011). C20-D3-vitamin A slows lipofuscin accumulation and electrophysiological retinal degeneration in a mouse model of Stargardt disease. J. Biol. Chem..

[24] Huang J., Possin D.E., Saari J.C. (2009). Localizations of visual cycle components in retinal pigment epithelium. Mol. Vis..

[25] Saari J.C., Nawrot M., Garwin G.G., Kennedy M.J., Hurley J.B., Ghyselinck N.B. (2002). Analysis of the visual cycle in cellular retinol-binding protein type I (CRBPI) knockout mice. Invest. Ophthalmol. Vis. Sci..

[26] Ghyselinck N.B., Bavik C., Sapin V., Mark M., Bonnier D., Hindelang C. (1999). Cellular retinol-binding protein I is essential for vitamin A homeostasis. EMBO J..

[27] Silvaroli J.A., Widjaja-Adhi M.A.K., Trischman T., Chelstowska S., Horwitz S., Banerjee S. (2019). Abnormal cannabidiol modulates vitamin A metabolism by acting as a competitive inhibitor of CRBP1. ACS Chem. Biol..

[28] Plau J., Morgan C.E., Fedorov Y., Banerjee S., Adams D.J., Blaner W.S. (2023). Discovery of nonretinoid inhibitors of CRBP1: structural and dynamic insights for ligand-binding mechanisms. ACS Chem. Biol..

[29] Chen Y., Okano K., Maeda T., Chauhan V., Golczak M., Maeda A. (2012). Mechanism of all-trans-retinal toxicity with implications for Stargardt disease and age-related macular degeneration. J. Biol. Chem..

[30] Maeda A., Maeda T., Golczak M., Palczewski K. (2008). Retinopathy in mice induced by disrupted all-trans-retinal clearance. J. Biol. Chem..

[31] Kohno H., Chen Y., Kevany B.M., Pearlman E., Miyagi M., Maeda T. (2013). Photoreceptor proteins initiate microglial activation via toll-like receptor 4 in retinal degeneration mediated by all-trans-retinal. J. Biol. Chem..

[32] LaVail M.M., Gorrin G.M., Repaci M.A. (1987). Strain differences in sensitivity to light-induced photoreceptor degeneration in albino mice. Curr. Eye Res..

[33] Lyubarsky A.L., Savchenko A.B., Morocco S.B., Daniele L.L., Redmond T.M., Pugh E.N. (2005). Mole quantity of RPE65 and its productivity in the generation of 11-cis-retinal from retinyl esters in the living mouse eye. Biochemistry.

[34] Grimm C., Reme C.E. (2013). Light damage as a model of retinal degeneration. Methods Mol. Biol..

[35] Oetting W.S., King R.A. (1999). Molecular basis of albinism: mutations and polymorphisms of pigmentation genes associated with albinism. Hum. Mutat..

[36] Maeda A., Maeda T., Golczak M., Chou S., Desai A., Hoppel C.L. (2009). Involvement of all-trans-retinal in acute light-induced retinopathy of mice. J. Biol. Chem..

[37] Kubota R., Boman N.L., David R., Mallikaarjun S., Patil S., Birch D. (2012). Safety and effect on rod function of ACU-4429, a novel small-molecule visual cycle modulator. Retina.

[38] Dobri N., Qin Q., Kong J., Yamamoto K., Liu Z., Moiseyev G. (2013). A1120, a nonretinoid RBP4 antagonist, inhibits formation of cytotoxic bisretinoids in the animal model of enhanced retinal lipofuscinogenesis. Invest. Ophthalmol. Vis. Sci..

[39] Charbel Issa P., Barnard A.R., Herrmann P., Washington I., MacLaren R.E. (2015). Rescue of the Stargardt phenotype in Abca4 knockout mice through inhibition of vitamin A dimerization. Proc. Natl. Acad. Sci. U. S. A..

[40] Batten M.L., Imanishi Y., Maeda T., Tu D.C., Moise A.R., Bronson D. (2004). Lecithin-retinol acyltransferase is essential for accumulation of all-trans-retinyl esters in the eye and in the liver. J. Biol. Chem..

[41] Moise A.R., Golczak M., Imanishi Y., Palczewski K. (2007). Topology and membrane association of lecithin: retinol acyltransferase. J. Biol. Chem..

[42] Golczak M., Sears A.E., Kiser P.D., Palczewski K. (2015). LRAT-specific domain facilitates vitamin A metabolism by domain swapping in HRASLS3. Nat. Chem. Biol..

[43] Zhang J., Dong Z., Mundla S.R., Hu X.E., Seibel W., Papoian R. (2015). Expansion of first-in-class drug candidates that sequester toxic all-trans-retinal and prevent light-induced retinal degeneration. Mol. Pharmacol..

[44] Marlhens F., Bareil C., Griffoin J.M., Zrenner E., Amalric P., Eliaou C. (1997). Mutations in RPE65 cause Leber's congenital amaurosis. Nat. Genet..

[45] Thompson D.A., Li Y., McHenry C.L., Carlson T.J., Ding X., Sieving P.A. (2001). Mutations in the gene encoding lecithin retinol acyltransferase are associated with early-onset severe retinal dystrophy. Nat. Genet..

[46] Seeliger M.W., Biesalski H.K., Wissinger B., Gollnick H., Gielen S., Frank J. (1999). Phenotype in retinol deficiency due to a hereditary defect in retinol binding protein synthesis. Invest. Ophthalmol. Vis. Sci..

[47] Maw M.A., Kennedy B., Knight A., Bridges R., Roth K.E., Mani E.J. (1997). Mutation of the gene encoding cellular retinaldehyde-binding protein in autosomal recessive retinitis pigmentosa. Nat. Genet..

[48] Furuhashi M., Hotamisligil G.S. (2008). Fatty acid-binding proteins: role in metabolic diseases and potential as drug targets. Nat. Rev. Drug Discov..

[49] Chen S., Pan Z., Liu M., Guo L., Jiang X., He G. (2024). Recent advances on small-molecule inhibitors of lipocalin-like proteins. J. Med. Chem..

[50] Parish C.A., Hashimoto M., Nakanishi K., Dillon J., Sparrow J. (1998). Isolation and one-step preparation of A2E and iso-A2E, fluorophores from human retinal pigment epithelium. Proc. Natl. Acad. Sci. U. S. A..

[51] Carter T.C., Falconer D.S. (1951). Stocks for detecting linkage in the mouse, and the theory of their design. J. Genet..

[52] Golczak M., Bereta G., Maeda A., Palczewski K. (2010). Molecular biology and analytical chemistry methods used to probe the retinoid cycle. Methods Mol. Biol..

