# Supplementary material for: Inactivation of cellular retinol-binding protein 1 protects against bis-retinoid accumulation and light-induced retinal degeneration in mice
Source: J Biol Chem. 2025 Jul 30;301(9):110538. doi: 10.1016/j.jbc.2025.110538 (PMC12398791; doi:10.1016/j.jbc.2025.110538)
Supplement: Supporting information [file mmc1.docx]

**Supplemental information**

**Inactivation of cellular retinol-binding protein 1 protects against bis-retinoid accumulation and light-induced retinal degeneration in mice**

Made Airanthi K. Widjaja-Adhi^1#^, Jaclyn Swigris^1^, Jacqueline Plau^1^, Chloe Chung^1^, Anna Walczak-Szeffer^1,2^, Beata Jastrzebska^1,3^, William S. Blaner^4^, and Marcin Golczak^1,3,#^

From the ^1^Department of Pharmacology, School of Medicine, Case Western Reserve University, Cleveland, OH; ^2^Department of Cell Cultures and Genomic Analysis, Medical University of Lodz, Lodz, Poland; ^3^Cleveland Center for Membrane and Structural Biology, School of Medicine, Case Western Reserve University, Cleveland, OH; ^4^Department of Medicine, College of Physicians and Surgeons, Columbia University, New York, NY.

**Running title:** Genetic validation of RBP1 as a pharmacological target

**Keywords:** retinol-binding protein 1, visual cycle, A2E, retinoids, retinal degeneration, RBP1

^#^To whom the correspondence should be addressed:

Marcin Golczak, Ph.D., Department of Pharmacology, School of Medicine, Case Western Reserve University, 10900 Euclid Ave, Cleveland, Ohio 44106, USA; Phone: 216–368–0302; E-mail: [mxg149@case.edu](mailto:mxg149@case.edu).

Made Airanthi K. Widjaja-Adhi, Ph.D., Department of Pharmacology, School of Medicine, Case Western Reserve University, 10900 Euclid Ave, Cleveland, Ohio 44106, USA; Phone: 216–368–0302; E-mail: nkw5@case.edu.

**Supplemental Table 1** – *Inhibitors of RBP1 used in this study.*

| **Inhibitor**  **abbreviation** | **PubChem**  **ID#** | **Hit2Lead**  **ID#** | **IUPAC name** | ***K_i_* (µM)** |
| --- | --- | --- | --- | --- |
| **abn-CBD** | 89949 | N/A | 4-[(1R,6R)-3-methyl-6-prop-1-en-2-ylcyclohex-2-en-1-yl]-5-pentylbenzene-1,3-diol | 0.067± 6.7 |
| **Z5H** | 25370031 | 28421637 | N-methyl-N-[[3-[1-(4-methylphenyl)cyclopentyl]-1,2,4-oxadiazol-5-yl]methyl]-1-thiophen-2-ylmethanamine | 9.0 ± 4.1 |
| **ZDF** | 42194644 | 73251475 | N-ethyl-N-[[3-[1-(4-methylphenyl)cyclopentyl]-1,2,4-oxadiazol-5-yl]methyl]-2-pyrazol-1-ylethanamine | 7.1 ± 2.7 |
| **ZDK** | 42094040 | 10035007 | 3-benzhydryl-5-[[4-(methoxymethyl)piperidin-1-yl]methyl]-1,2,4-oxadiazole | 8.3 ± 2.8 |

**Supplemental Table 2** – *Summary of conditions used for the genotyping of the genetically modified mice.*

***Rbp1* genotyping**

| Component | Details |
| --- | --- |
| **Primers** | WT: VI35 – AAA AAT GGA AAG GCA AGG CAC AGA C  Mutant: NM9 – GCC TTC TAT CGC CTT CTT GAC GAG TTC TTC  Common: UE79 – GCA CTT GCG GTC GTC TAT GC |
| **PCR Mix** | 0.5 μL DNA, 0.75 μL 50 mM MgCl₂, 0.5 μL 10 mM dNTPs, 0.5 μL 3-primer mix (5 μL UE79, 5 μL NM9, 2 μL VI35 in 50 μL), 0.1 μL Platinum Taq |
| **Cycling Conditions** | Initial denaturation: 94°C, 2 min  Touchdown: 10 cycles (94°C 15s, 65–60°C -0.5°C/cycle 30s, 68°C 20s)  Stage 2: 38 cycles (94°C 15s, 61°C 30s, 72°C 20s) |
| **Amplicon Sizes** | WT: 338 bp; Mutant: 593 bp |

***Abca4* Genotyping**

| Component | Details |
| --- | --- |
| **Primers** | CommonF: CTT TGC TAC TAT CCT GCT GAG TTT G  WTR2: CTT TTG TGA GGG AAA GGA GAC AG  MutR2: CCG CTT CCT CGT GCT TTA C |
| **PCR Mix** | 0.5 μL DNA, 0.75 μL 50 mM MgCl₂, 0.5 μL 10 mM dNTPs, 1.25 μL DMSO, 0.5 μL 3-primer mix (10 μM), 0.1 μL Platinum Taq |
| **Cycling Conditions** | Same as Rbp1 except Stage 2: 30 cycles (94°C 15s, 60°C 30s, 72°C 20s) |
| **Amplicon Sizes** | WT: 418 bp; Mutant: 525 bp |

***Rdh8* genotyping**

| Component | Details |
| --- | --- |
| **Primers** | WT: 32345 – CAC AAC ATC CCA GCA CTC TG  Mutant: oIMR2088 – AGA CTG CCT TGG GAA AAG CG  Common: 32346 – ACT CCG CCT TGG AAA CCT G |
| **PCR Mix** | 0.5 μL DNA, 0.75 μL 50 mM MgCl₂, 0.5 μL 10 mM dNTPs, 1.25 μL DMSO, 0.5 μL 3-primer mix (5 μL 32346, 4 μL oIMR2088, 5 μL 32345 in 50 μL), 0.1 μL Platinum Taq |
| **Cycling Conditions** | Same as Rbp1 except Stage 2: 28 cycles (94°C 15s, 60°C 30s, 72°C 20s) |
| **Amplicon Sizes** | WT: 300 bp; Mutant: 400 bp |

***rd8* Mutation detection**

| Component | Details |
| --- | --- |
| **Primers** | WT Mix: RD8C2 – GGT GTA TCC AGG CTC ACA C +RD8WT – GAA GAC AGC TAC AGT TCT TAT CGG  Mutant Mix: RD8F3450 – CTG TCT GAG CAC AAT AGA GAT TGG + RD8 KO – CGA GAG ACA GGC ACA CCA TA  Internal Control: oIMR7338 – CTA GGC CAC AGA ATT GAA AGA TCT and oIMR7339 – GTA GGT GGA AAT TCT AGC ATC ATC C |
| **PCR Mix** | 0.5 μL DNA, 0.75 μL 50 mM MgCl₂, 0.5 μL 10 mM dNTPs, 0.75 μL WT or Mutant primer mix, 0.5 μL internal control primers, 0.1 μL Platinum Taq |
| **Cycling Conditions** | 94°C for 2 min, then 41 cycles: 94°C 15s, 66°C 10s, 72°C 20s |
| **Amplicon Sizes** | WT: 159 bp; Mutant: 216 bp; Internal Control: 324 bp |

***Rpe65* L450M variant genotyping**

| Component | Details |
| --- | --- |
| **Primers** | Forward: ACC AGA AAT TTG GAG GGA AAC  Reverse: CCC TTC CAT TCA GAG CTT CA |
| **PCR Mix** | 0.5 μL DNA, 0.75 μL 50 mM MgCl₂, 0.5 μL 10 mM dNTPs, 0.5 μL 10 μM primer mix, 0.1 μL Platinum Taq |
| **Cycling Conditions** | Initial: 94°C for 2 min; 36 cycles: 94°C 15s, 60°C 20s, 72°C 20s |
| **Post-PCR** | Digest 5 μL PCR product with MwoI (5 μL mix: 1 μL buffer, 0.4 μL enzyme, 3.6 μL H₂O); incubate 2 h at 60°C |
| **Amplicon Sizes** | WT: 380 + 180 bp (cut); Mutant: 560 bp (uncut) |


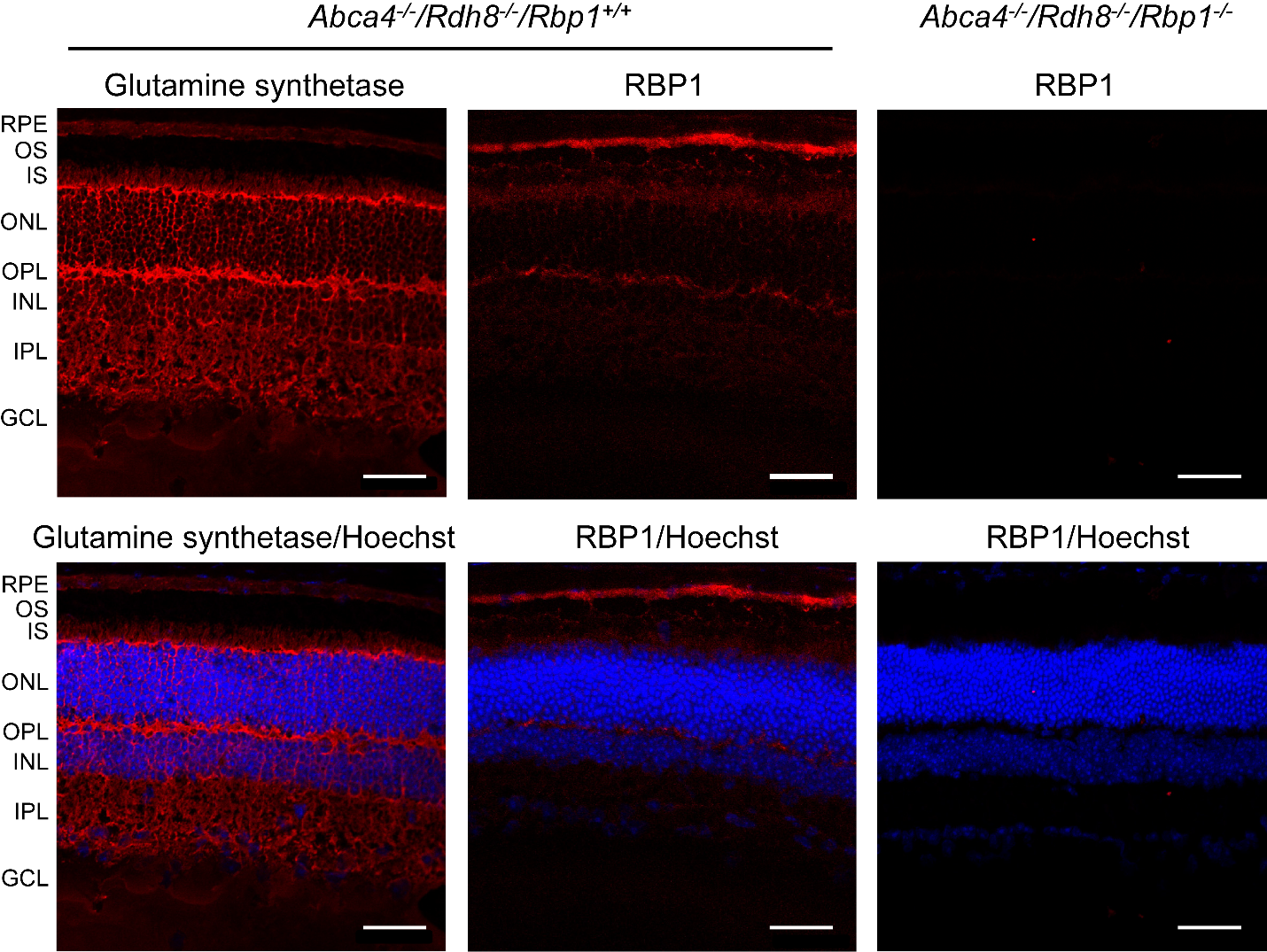


**Supplemental Figure 1** – *Immunofluorescence-based analysis of RBP1 distribution in mouse retina.* RBP1 staining is observed in the RPE of *Abca4^-/-^/Rdh8^-/-^* mice. In contrast, there is no definitive evidence of RBP1 expression in the neuronal retina, aside from a slight increase in background fluorescence in the outer plexiform layer. When contrast gain is increased, faint staining appears in a region corresponding to the apical processes of Müller cells; however, this pattern is distinct from that of glutamine synthetase, a known marker of Müller glia. No immunofluorescence signal was detected in *Rbp1^-/-^* mice on the *Abca4^-/-^/Rdh8^-/-^* genetic background. Scale bar: 50 μm.


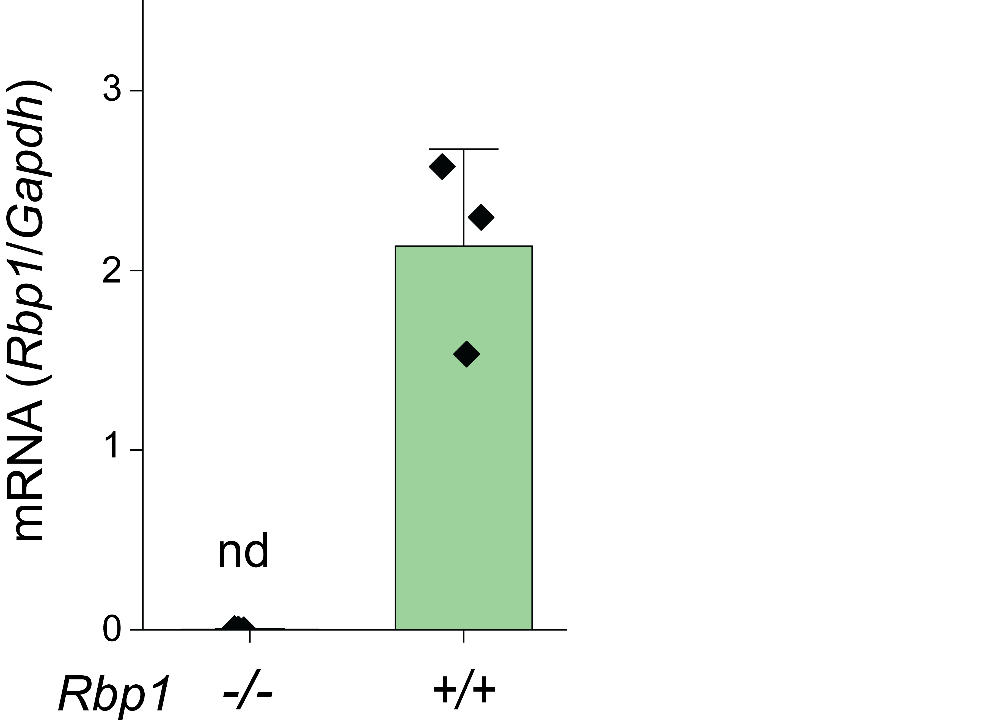


**Supplemental Figure 2** – *Quantitative RT-PCR analysis of Rbp1 transcript expression in mouse eyes.* Total mRNA was isolated from whole-eye homogenates of *Abca4^-/-^/Rdh8^-/-^* and *Abca4^-/-^/Rdh8^-/-^/Rbp1^-/-^* mice and subjected to quantitative reverse transcription PCR (qRT-PCR) to detect *Rbp1* transcript levels. The experiment was independently repeated three times using mRNA isolated from different biological replicates. A robust amplification signal was observed in *Abca4^-/-^/Rdh8^-/-^* mice, while no detectable (nd) *Rbp1* signal was present in samples derived from RBP1-deficient animals, confirming the successful disruption of *Rbp1* expression in the *Abca4^-/-^/Rdh8^-/-^/Rbp1^-/-^* mouse line.


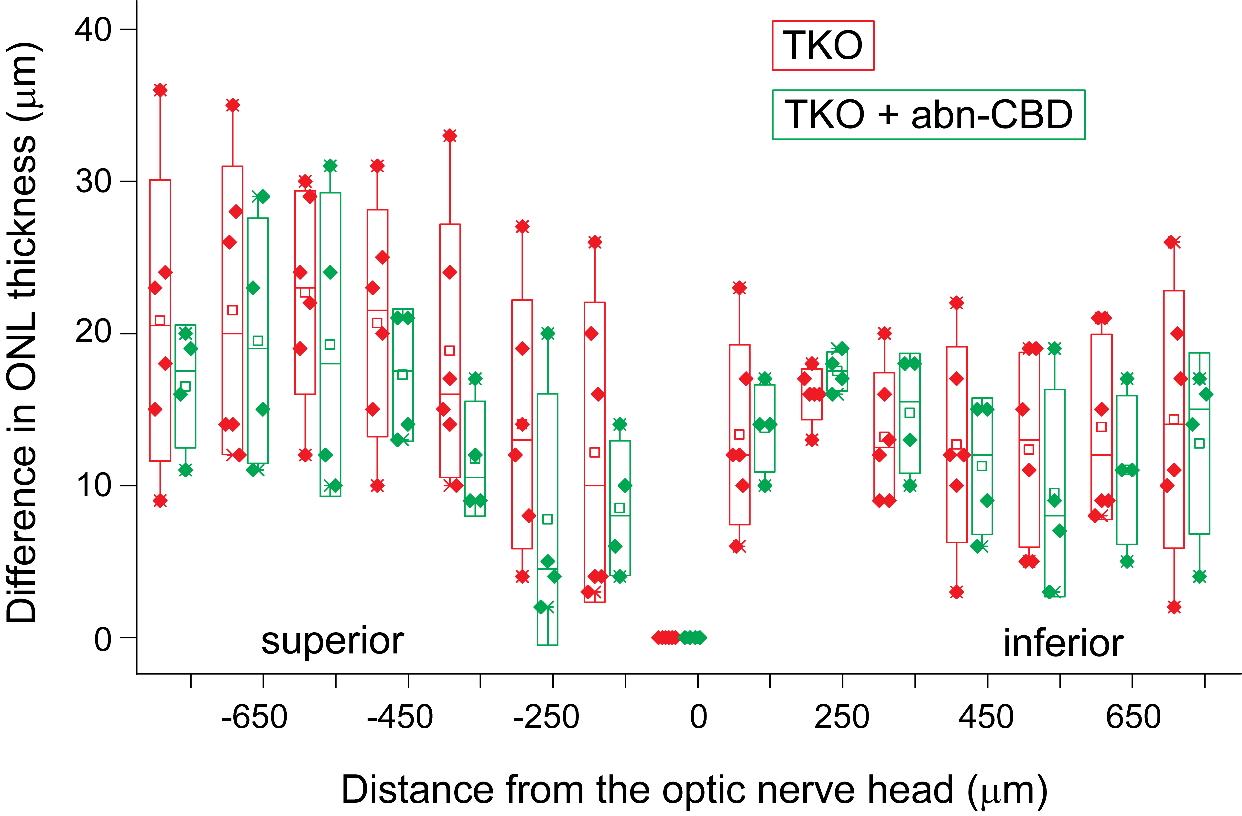


**Supplemental Figure 3** – *abn-CBD exerts its retinal protective effect via interaction with RBP1.* *Abca4^-/-^/Rdh8^-/-^/Rbp1^-/-^* (TKO) mice were treated with either abn-CBD (30 mg/kg) or vehicle (DMSO) and subjected to light-induced retinal stress, alongside a control group of *Abca4^-/-^/Rdh8^-/-^* mice. The data represent the difference in ONL thickness between *Abca4^-/-^/Rdh8^-/-^* (retinal damage control) and *Abca4^-/-^/Rdh8^-/-^/Rbp1^-/-^* mice treated with abn-CBD (green) or DMSO (red). The absence of statistically significant differences at any distance from the optic nerve indicates that abn-CBD treatment does not confer additional protection in the absence of RBP1.


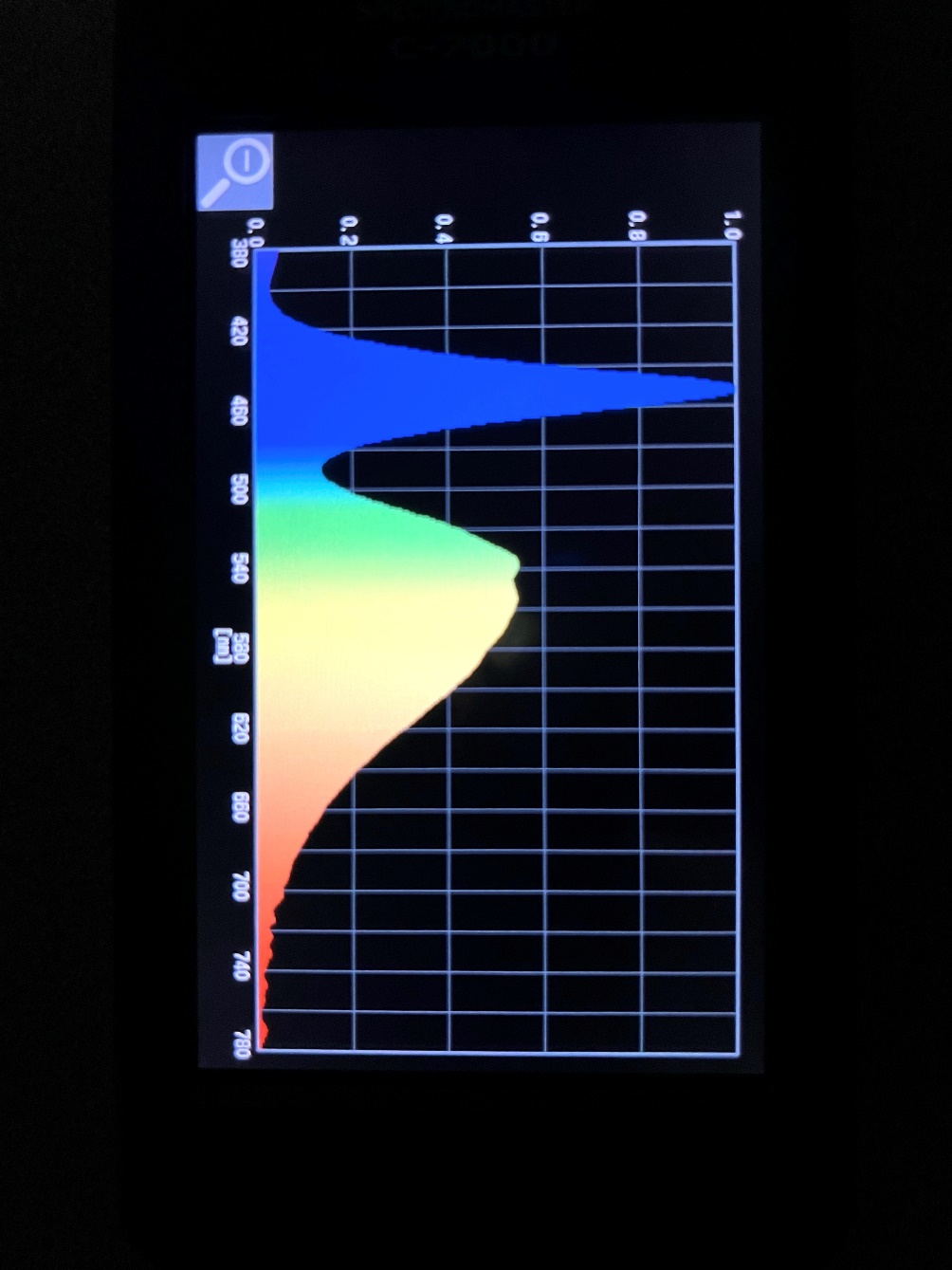


**Normalized intensity**

**Wavelength (nm)**

**Supplemental Figure 4** – *Spectrum of light emitted by the lamp used to inflict retinal damage in the Abca4^-/-^/Rdh8^-/-^ and Balb/cJ mouse models.*





**Supplemental Figure 5** – *Efficiency of light-induced retinal damage in Abca4^−/−^Rdh8^−/−^ mice.* The mice at 8-12 weeks of age were exposed to retinal-damaging light. The excess of the retinal insult was examined by OCT 5 days post-light exposure and quantified as the thickness of the ONL. Black and white triangles represent data for pre-bleach male (n = 10) and female (n = 6), while circles correspond to post-light damage (● – male, n = 22; ○ – female, n = 11). No statistically significant differences in light-induced retinal degeneration were observed between males and females at any distance from the optic nerve. Data are presented as mean ± S.D.
